# Pathoadaptation of the passerine-associated *Salmonella enterica* serovar Typhimurium lineage to the avian host

**DOI:** 10.1371/journal.ppat.1009451

**Published:** 2021-03-19

**Authors:** Emiliano Cohen, Shalevet Azriel, Oren Auster, Adiv Gal, Carmel Zitronblat, Svetlana Mikhlin, Felix Scharte, Michael Hensel, Galia Rahav, Ohad Gal-Mor

**Affiliations:** 1 The Infectious Diseases Research Laboratory, Sheba Medical Center, Tel-Hashomer, Israel; 2 Sackler Faculty of Medicine, Tel Aviv University, Tel Aviv, Israel; 3 Department of Clinical Microbiology and Immunology, Tel Aviv University, Tel Aviv, Israel; 4 Faculty of Sciences, Kibbutzim College, Tel-Aviv Israel; 5 Carmel Veterinary Clinic, Jerusalem, Israel; 6 Biovac Ltd., Or-Akiva, Israel; 7 Abteilung Mikrobiologie, Universität Osnabrück, Osnabrück, Germany; University of California Davis School of Medicine, UNITED STATES

## Abstract

*Salmonella enterica* is a diverse bacterial pathogen and a primary cause of human and animal infections. While many *S*. *enterica* serovars present a broad host-specificity, several specialized pathotypes have been adapted to colonize and cause disease in one or limited numbers of host species. The underlying mechanisms defining *Salmonella* host-specificity are far from understood. Here, we present genetic analysis, phenotypic characterization and virulence profiling of a monophasic *S*. *enterica* serovar Typhimurium strain that was isolated from several wild sparrows in Israel. Whole genome sequencing and complete assembly of its genome demonstrate a unique genetic signature that includes the integration of the BTP1 prophage, loss of the virulence plasmid, pSLT and pseudogene accumulation in multiple T3SS-2 effectors (*sseJ*, *steC*, *gogB*, *sseK2*, and *sseK3*), catalase (*katE*), tetrathionate respiration (*ttrB*) and several adhesion/ colonization factors (*lpfD*, *fimH*, *bigA*, *ratB*, *siiC* and *siiE*) encoded genes. Correspondingly, this strain demonstrates impaired biofilm formation, intolerance to oxidative stress and compromised intracellular replication within non-phagocytic host cells. Moreover, while this strain showed attenuated pathogenicity in the mouse, it was highly virulent and caused an inflammatory disease in an avian host. Overall, our findings demonstrate a unique phenotypic profile and genetic makeup of an overlooked *S*. Typhimurium sparrow-associated lineage and present distinct genetic signatures that are likely to contribute to its pathoadaptation to passerine birds.

## Author summary

During *Salmonella enteric*a evolution, many different ecological niches have been effectively occupied by this highly diverse bacterial pathogen. While many *S*. *enterica* serovars successfully maintained their ability to infect and colonize in a wide-array of host species, a few biotypes have evolved to colonize and cause a disease in only one or a small group of hosts. The evolutionary dynamic and the mechanisms shaping the host-specificity of *Salmonella* adapted strains are important to better understand *Salmonella* pathogenicity and its ecology, but still not fully understood. Here, we report genetic and phenotypic characterization of a *S*. Typhimurium strain that was isolated from several wild sparrows in Israel. This strain presented unique phenotypic profile that included impaired biofilm formation, high sensitivity to oxidative stress and reduced intracellular replication in non-phagocytic cells. In addition, while this strain was able to cause high inflammatory disease in an avian host, it was highly attenuated in the mouse model. Genome analysis identified that specific genetic signatures found in the sparrow strain are more frequently associated with poultry isolates than clinical isolates of *S*. Typhimurium. These genetic features are expected to accumulatively contribute toward the adaptation of this strain to birds.

## Introduction

Bacteria belonging to the species *Salmonella enterica* (*S*. *enterica*) are a leading cause of foodborne diseases and a common source of human and animal infections. Based on the somatic (O), flagella (H), and capsular (Vi) antigenic determinants, more than 2,600 distinct serovars have been defined so far in this highly diverse species [1,2]. The clinical symptoms elicited by serovars of *S*. *enterica* range from self-limiting gastroenteritis to systemic diseases, including bacteremia caused by invasive nontyphoidal *Salmonella* (iNTS) and enteric fever [3].

*S*. *enterica* serovars could be classified according to their host-specificity into three groups. Most of the serovars, like *S*. Typhimurium or *S*. Enteritidis are generalists, present a broad-host range and can infect a diverse array of host species. A second group is the host-adapted serovars that usually cause disease in one particular host species, but occasionally infect other hosts, including humans. Examples for such serovars are *S*. Choleraesuis (swine adapted), *S*. Dublin (bovine adapted) or *S*. Abortusovis (sheep adapted) [4]. The third group of serovars are the ones that are fully host-specific (or host-restricted) and are capable to infect only one host species, like *S*. Typhi and *S*. Paratyphi A that infect and cause a systemic life-threatening enteric fever, only in humans and higher primates [3,5]. Similarly, other serovars have adapted to a specific non-human host, such as *S*. Gallinarum or *S*. Pullorum to the avian host, in which they cause a systemic invasive disease known as fowl typhoid and pullorum disease, respectively [6].

Intracellular replication is one of the hallmarks of *S*. *enterica* and is tightly linked to its ability to cause systemic infection [7,8]. This phenotype is largely dependent on a functional type three secretion system (T3SS) encoded on the *Salmonella* pathogenicity island (SPI)-2 and its associated translocated effectors, involved in manipulating the unfavorable intracellular environment and support *Salmonella* survival and replication within host cells [9]. Additionally, SPI-2 and a subset of its cognate effectors were also shown to interfere with innate immune responses [10] and contribute to intracellular persistence of *Salmonella* inside fibroblasts [11].

*S*. Typhimurium is one of the most prevalent serovars responsible for animal and human infections worldwide. *S*. Typhimurium variant with the antigenic formula 1,4,[5],12:i:- is known as a monophasic *S*. Typhimurium (MpSTM), which does not express the phase 2 flagellar antigen (FljB) [12]. Multiple clonal lineages of MpSTM are circulating globally, and many of them are multidrug resistant that were linked to reoccurring foodborne outbreaks, causing a significant epidemic health risk [13,14].

Even though *S*. Typhimurium is typically a generalist serovar, some specific phage types are commonly associated with a specific host and present a distinct pathogenicity. For example, *S*. Typhimurium of definitive phage types (DT) 8, 2 and 56 are frequently associated with infections in ducks, feral pigeon, and passerine birds, respectively [15–18]. Similarly, specific clonal group of *S*. Typhimurium known as sequence type (ST) 313 is responsible for an invasive salmonellosis in humans in sub-Saharan Africa, manifesting as bacteremia and meningitis [19] and for systemic infections in chickens [20]. Thus, these observations suggest that certain pathotypes of *S*. Typhimurium have evolved from broad-host range lineages to host-restricted stains, while the underlying mechanisms involved in *Salmonella* host-specificity are still not fully understood.

Passerine salmonellosis is a known disease caused by *S*. Typhimurium that affects mainly species from the finch (Fringillidae) and sparrow (Passeridae) families, including the house sparrow [21,22]. *S*. Typhimurium strains of phage types DT56 and DT40 were previously shown to be associated with passerine birds, but since they occasionally infect humans, livestock and companion animals, their host-specificity and virulence profile remained somewhat elusive [17,23].

Here, we report systematic genomic, phenotypic and virulence studies of a passerine-adapted MpSTM strain that was isolated from wild sparrows in Israel. Our data demonstrate the different pathogenicity of this lineage in mammalian and avian hosts and present the genetic makeup, which likely facilitates this phenotypic pattern and host adaptation.

## Results

### Isolation of MpSTM clonal isolates from wild sparrows

Screening sedentary and migratory bird populations in Israel for *Salmonella* carriage, has led to the identification of two House Sparrows (*Passer domesticus*) and two Spanish Sparrows (*Passer hispaniolensis*) infected with *S*. *enterica*. All four *Salmonella*-infected birds were sampled in the same region near Ga’ash, located in the coastal plain to the north of Tel-Aviv, Israel (Latitude 32.2290805472; Longitude 34.8207548408). Serotyping of these isolates (designated AB42049, AB42052, AB42086, and AB42142) showed the same uncommon antigenic formula 4,12:-:1,2, corresponding to MpSTM that does not express the first phase of the flagellum antigen (i) of *S*. Typhimurium. While MpSTM that do not express the second flagellum phase are relatively prevalent, the serotype 4,12:-:1,2 is very rare in Israel. In the last 3 years, out of 1,300 *S*. Typhimurium isolates that were characterized at the National *Salmonella* Reference Center, only 3 isolates (two clinical and one from poultry) were serotyped as 4,12:-:1,2.

**Fig 1:**
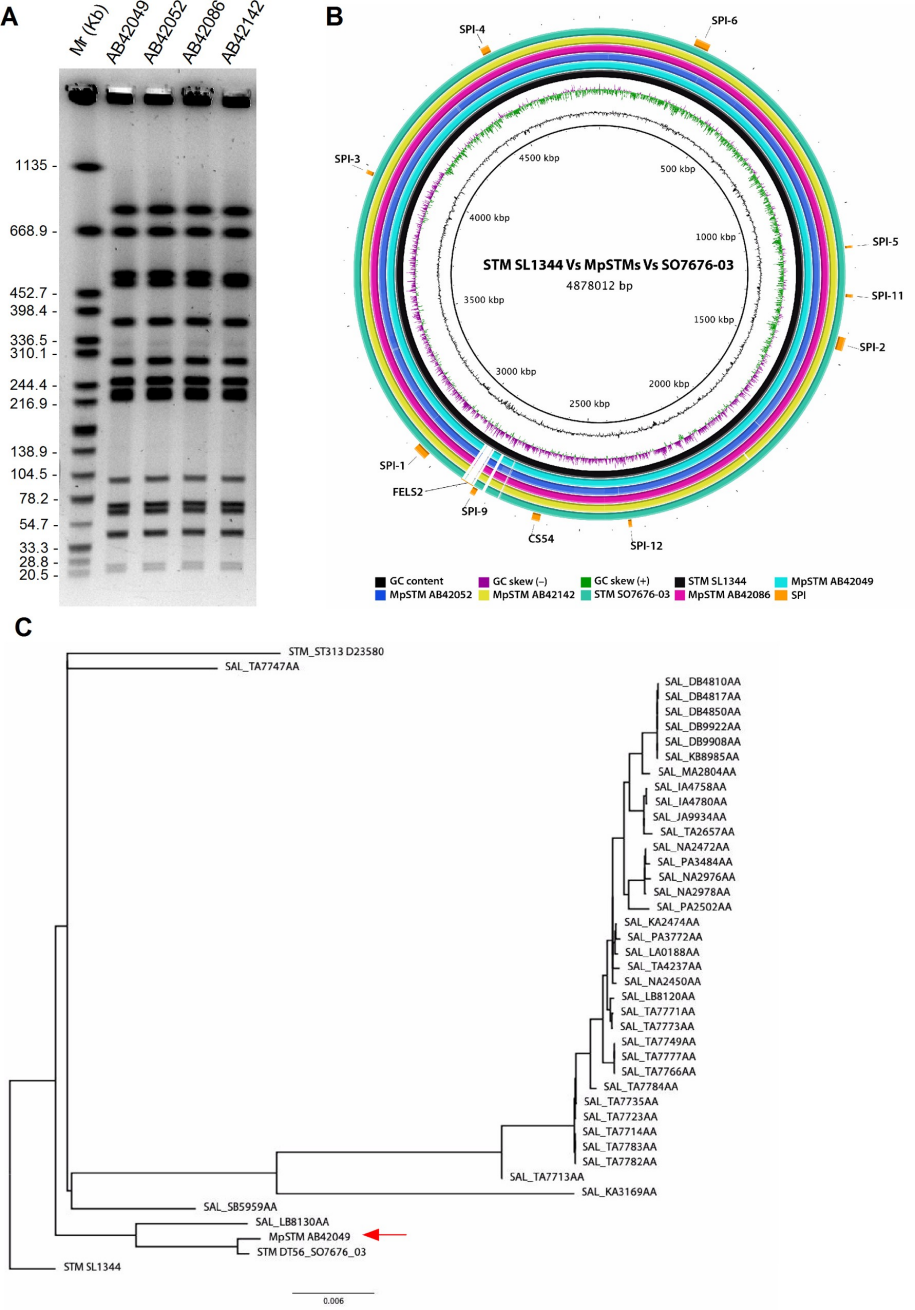
Identification of a clonal *S*. Typhimurium strain associated with wild sparrows. (**A**) The genetic similarity of four sparrow isolates (AB42049, AB42052, AB42086, and AB42142), which were isolated in Israel at 2015 was analyzed by PFGE following digest with the restriction enzyme, XbaI. (**B**) The genome assemblies of the four sparrow-associated isolates and the sparrow strain isolated in England at 2005 (SO7676-03) were compared to the published genome of the reference *S*. Typhimurium SL1344 (black ring) strain using the BRIG tool. The distribution and position of SPIs are shown in orange boxes and absence of genomic elements, in relation to the SL1344 genome is indicated by white gaps. (**C**) A phylogenetic tree of isolate AB42049 (highlighted by a red arrow) in relation to *S*. Typhimurium strains SL1344, SO7676-03, ST313 (D23580) and additional 38 monophasic *S*. Typhimurium isolated from an avian/ poultry source registered in Enterobase. The tree was based on 4758 SNPs in their core genome (4525629 bp) and the SL1344 genome was used as the reference.

Pulsed field gel electrophoresis (Fig 1A) and whole genome sequencing (Fig 1B) have indicated clonality with high genetic similarity between all four isolates. Moreover, their genome exhibited very high sequence similarity to a *S*. Typhimurium DT56 passerine isolate (strain SO7676-03; Bioproject PRJEB34599) that was isolated at northern England at 2005 [16,24] (Fig 1C). Variant calling between SO7676-03 and AB42049 indicated the presence of 141 variations (including 115 SNPs and 24 indels of 1–4 nucleotides; S1 Table), suggesting close phylogenetic relationship and that this *S*. Typhimurium lineage is a passerine-associated strain.

### Genetic characterization of the sparrows-associated strain

To generate a gap-free complete assembly of this MpSTM strain, we have performed hybrid genome assembly of isolate AB42049, combining both short (Illumina) and long (Oxford Nanopore) reads. The assembled genome of AB42049 is 4,862,477 bp long, has a G+C content of 52.2% and predicted to encode 4527 CDSs. The AB42049 genome contains a single chromosome with no plasmids. Noteworthy, the lack of the pSLT virulence plasmid, often associated with *S*. Typhimurium isolates [25]. Sequence analysis indicated the presence of SPIs 1–5, 9, 11, 12, CS54, as well as the integration of prophages Fels-1, Gifsy-1, Gifsy-2 and ST64B, as in *S*. Typhimurium SL1344, but the absence of the Fels-2 prophage (Fig 1B). Interestingly, this sparrow isolate also harbors the BTP1 prophage (S1 Fig), previously found in the invasive *S*. Typhimurium ST313 strain [26].

In addition to pSLT absence, pseudogene analysis identified the presence of at least 67 inactivated genes, many of which are involved in *Salmonella* metabolism and pathogenicity (S2 Table). An intriguing mutation was identified in the tetrathionate anaerobic respiration pathway. *S*. Typhimurium exploits tetrathionate respiration encoded by the intact *ttrRS/ttrBCA* gene cluster [27] to gain metabolic advantage over the gut microbiota [28]. Here we found that the tetrathionate metabolic gene, *ttrB*, is inactive in the AB42049 genome. Inactivation of this pathway prevents the use of tetrathionate as terminal electron acceptors under reduced oxygen conditions and possibly impaired competition with the microbiome during intestinal colonization.

Pseudogene formation in multiple host adhesion factors were also evident. Two fimbrial genes including *lpfD* (encoding the tip adhesin of the Lpf fimbriae) and *fimH* (encoding the adhesin of the mannose-specific type 1 fimbriae) are inactive in the Sparrow MpSTM strain. Likewise, four nonfimbrial adhesion genes including *bigA*, encoding autotransporter adhesin; *ratB*, encoded on the CS54 island and required for optimal colonization in the mouse cecum [29]; and two SPI-4 encoded genes *siiC* and *siiE*, which contribute to *Salmonella* colonization in bovine and mice [30,31] are all pseudogenes as well.

Moreover, the hydrogen peroxide scavenger, catalase encoded gene *katE* and five T3SS-2 effector genes including *sseJ* (regulates SCV membrane dynamics), *steC* (phosphorylates MAP kinases to induce actin formation around SCVs and regulates intracellular replication), *sseK2* and *sseK3* (inhibit NF-κB signaling) and *gogB* (encoding E3 ubiquitin ligase that inhibits NF-κB signaling) (reviewed in [32]) were found to be pseudogenes. All of these genes contain nonsense or frame-shift mutations that were confirmed by targeted Sanger sequencing (S2 Fig).

Overall, this pattern of genome degradation is likely to profoundly affect the virulence of this strain and its interactions with the host(s). To address that, we next performed, phenotypic and virulence comparisons with the closely related *S*. Typhimurium reference strain SL1344.

### The sparrow MpSTM is susceptible to oxidative stress and does not form biofilm

**Fig 2:**
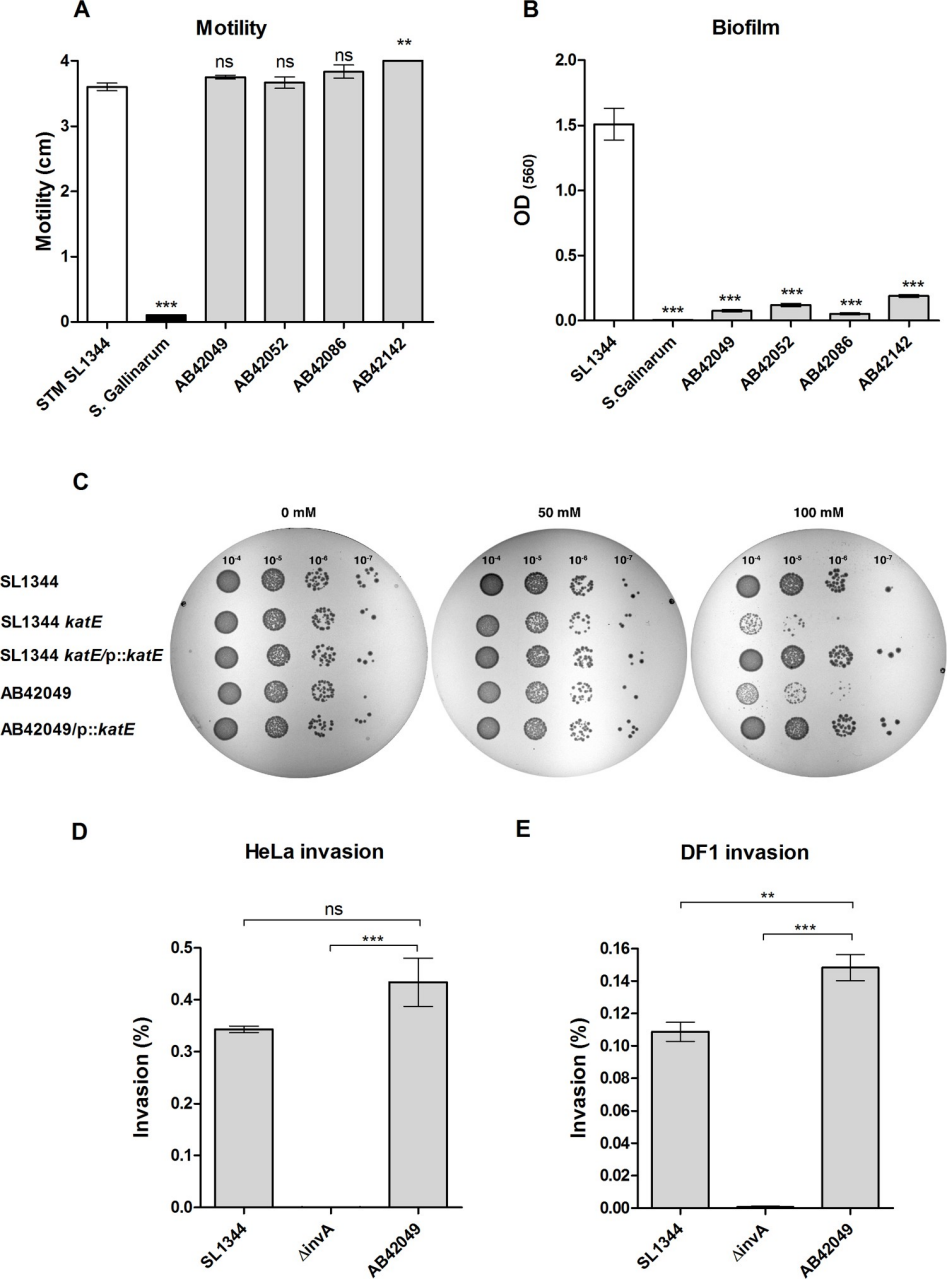
The sparrow-associated strain is impaired in biofilm formation and intolerant to oxidative stress. **(A**) The motility of the four sparrow MpSTM isolates was determined on soft agar plates and compared to the motility of *S*. Typhimurium SL1344 (positive control) and *S*. Gallinarum (negative control). The diameter of the motility ring after 5 h at 37°C is shown. The bars show the mean of three independent biological repeats and the standard error of the mean (SEM) is indicated by the error bars. (**B**) The ability of the above strains to form biofilm was determined by Crystal Violet staining of static cultures that were incubated for 96 h at 28°C in rich medium in the absence of sodium chloride (biofilm induced conditions). The amount of biofilm was evaluated by the absorbance of the stain at OD_560_. The bars show the mean of six biological repeats and the SEM is indicated by the error bars. (**C**) *S*. Typhimurium SL1344, SL1344Δ*katE*, AB42049, and SL1344Δ*katE* and AB42049 expressing the gene *katE* form a plasmid (pWSK29) were grown for overnight in LB medium to the stationary phase. Oxidative stress tolerance was tested by exposing these cultures to 0, 50 and 100 mM hydrogen peroxide for 20 min. Drops (10 μl) from serial dilutions were plated on LB-agar plates and imaged after an overnight incubation at 37°C. (**D-E**) The invasion of *S*. Typhimurium AB42049, SL1344, and its isogenic strain harboring a null deletion in *invA* was tested by the gentamicin protection assay in HeLa human epithelial cells (**D**) and in DF-1 chicken fibroblasts (**E**). Invasion was determined as the percentage of intracellular bacteria at 2 h post infection (p.i.), from the infecting inoculum. The bars show the mean of four biological repeats and the SEM is indicated by the error bars. 1–Way ANOVA with Dunnett’s Multiple Comparison Test was used to determine statistical difference. ns, not significant; **, *P*<0.01; ***, *P*<0.001.

Motility on soft agar plates showed that all four sparrow isolates are highly motile, demonstrating comparable motility to the one of *S*. Typhimurium SL1344 (Fig 2A). As a negative control, we included the host-specific *S*. Gallinarum that is non-motile [33]. These results indicated that the lack of the flagellum first phase antigen expression does not affect the motility of this strain. In contrast, all four isolates formed biofilm to a much lower extent than *S*. Typhimurium SL1344 and presented comparable biofilm formation levels as *S*. Gallinarum (Fig 2B).

Identification of a nonsense mutation in *katE*, encoding the stationary-phase catalase suggested that this strain is susceptible to hydrogen peroxide. To test this, we exposed stationary-phase cultures to varying concentrations of H_2_O_2_ and determined their survival. As a control, we constructed a null *katE* deletion in *S*. Typhimurium SL1344 background. These experiments showed that while *S*. Typhimurium SL1344 are resistant to short (20 min) exposure of 100 mM hydrogen peroxide, its *katE* mutant and the sparrow MpSTM strain are severely susceptible to these oxidative stress conditions. Introducing the *S*. Typhimurium *katE* gene, under its native promoter into *S*. Typhimurium Δ*katE* or the sparrow MpSTM strain in trans, rescued their hydrogen peroxide susceptibility and conferred tolerance at similar levels as *S*. Typhimurium SL1344 (Fig 2C).

Next, we examined the ability of the sparrow MpSTM to invade host cells. *S*. Typhimurium SL1344 and its isogeneic *invA* mutant strain, impaired in non-phagocytic cell invasion were used as comparative controls. Invasion assays into human epithelial cells (HeLa; Fig 2D) and chicken fibroblasts (DF-1; Fig 2E) demonstrated that despite gene inactivation in several adhesins including *siiC* and *siiE*, involved in adhesion and apical invasion into enterocytes [34,35], the sparrow MpSTM was able to successfully invade into non-phagocytic host cells *in-vitro*. Worthy of note, too, is that in the chicken fibroblasts the sparrow MpSTM consistently presented moderately elevated invasion than SL1344.

These results indicated that in spite of the monophasic nature of this strain and gene inactivation in several attachment factors, the sparrow MpSTM presents normal motility and host cells invasion compared to *S*. Typhimurium SL1344, but is impaired in biofilm formation and hydrogen peroxide tolerance.

### The sparrow MpSTM is impaired in intracellular replication

**Fig 3:**
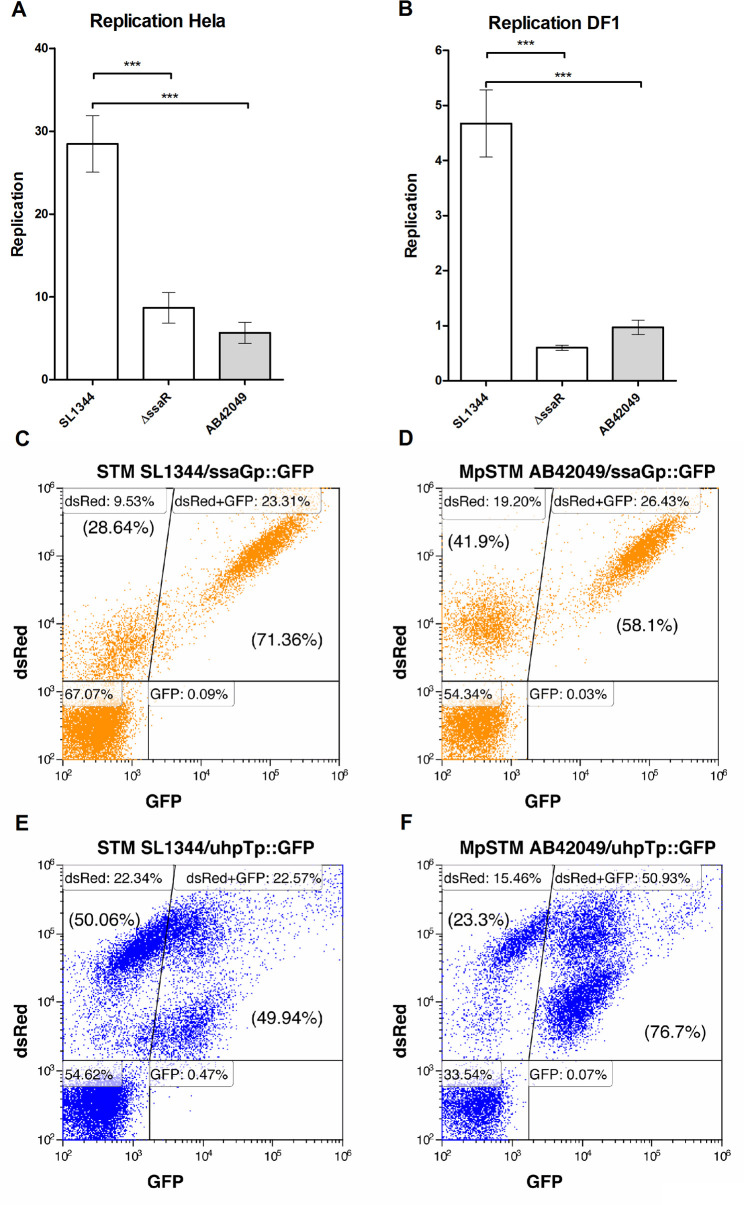
The *S*. Typhimurium sparrow-adapted strain is impaired in intracellular replication. Intracellular replication of *S*. Typhimurium AB42049, SL1344, and its isogenic strain harboring null deletion in *ssaR* was tested by the gentamicin protection assay in HeLa human epithelial cells (**A**) and in DF-1 chicken fibroblasts (**B**). Intracellular replication was determined by the ratio between the number of intracellular bacteria at 24 h p.i. relative to their number at 2 h p.i. using the gentamicin protection assay and direct plating on LB-agar plates. The bars show the mean of four biological repeats and the SEM is indicated by the error bars. 1–Way ANOVA with Dunnett’s Multiple Comparison Test was used to determine statistical difference. ***, *P*<0.001. (**C-E**) DF-1 chicken fibroblasts were infected with *S*. Typhimurium AB42049S and SL1344 harboring the reporter plasmids p3776 (P_*ssaG*_::sfGFP) and p4889 (P_*uhpT*_::sfGFP), indicating the localization of the bacteria within the SCV and the cytoplasm, respectively. 8 h p.i., flow cytometry of 1×10^4^ infected cells (DsRed-positive cells) was applied to determine the intracellular localization of these strains.

We next tested intracellular replication of AB42049 in human HeLa epithelial cells and avian DF-1 fibroblasts. Interestingly, in contrast to the normal invasion of this strain, intracellular replication in HeLa (Fig 3A) and in DF-1 cells (Fig 3B) was significantly reduced and comparable to the one of *S*. Typhimurium *ssaR* strain that possess a nonfunctional T3SS-2.

To follow further the intracellular fate of the sparrow MpSTM strain, we introduced two dual color reporter plasmids to AB42049 and SL1344 strains. The first system contains a Superfolder GFP (sfGFP) under the promoter of the SPI-2 gene *ssaG* (P_*ssaG*_::sfGFP). This promoter is induced when *Salmonella* is within the SCV [36]. The second reporter system contains the sfGFP under the expression of the *uhpT* promoter (P_*uhpT*_::sfGFP), which is induced by glucose 6-phosphate, found in the host cell cytoplasm [37]. Both constructs constantly express red fluorescent protein under the EM7 promoter that was used to sort for *Salmonella* infected cells. DF-1 cells that were infected with *S*. Typhimurium SL1344 or AB42049 carrying these reporter plasmids demonstrated moderately higher infection rate of AB42049 compared to STM SL1344, as was also observed by the gentamicin protection assay (Fig 2E). This difference could be seen by the higher frequency of DsRed -positive cells in AB42049 infected cells [26.43+19.20 = 45.63 (Fig 3D) and 50.93+15.46 = 66.39 (Fig 3F)] vs. *S*. Typhimurium infected cells [23.31+9.53 = 32.84 (Fig 3C) and 22.57+22.34 = 44.91 (Fig 3E)] for the P_*ssaG*_::sfGFP and P_*uhpT*_::sfGFP reporter plasmids, respectively. Moreover, this reporter system indicated that while higher percentage of SCV-containing salmonellae was found for *S*. Typhimurium (71.36%; Fig 3C) than AB42049 (58.1%; Fig 3D), higher frequency of cytoplasmic bacteria were reported for AB42049 (76.7%; Fig 3F) compared to *S*. Typhimurium (49.94%; Fig 3E). Collectively, these data indicate that AB42049 does not replicate well within non-phagocytic host cells and tend to localize at higher frequency in the cytoplasm of DF-1 cells rather than the SCV, compared to STM SL1344.

### The sparrow MpSTM and *S*. Typhimurium express similar levels of SPI-2 genes during host cells infection

To test whether the inability of the sparrow-MpSTM strain to replicate well intracellularly is due to low expression of SPI-2 genes, we examined the expression of several T3SS-2 genes in LB culture vs. their expression during intracellular infection. As shown in S3A Fig, the expression of *ssaR*, *ssaV* (structural T3SS-2 genes), *ssrB* (SPI-2 regultor), *sseD* (T3SS-2 tranlocon gene), *sifA* and *sseG* (T3SS-2 effectors) were induced by about 100 to 1000-fold during DF-1 infection by AB42049 compared to their expression, during growth in rich LB medium. Moreover, we were able to demonstrate that during intracellular infection, AB42049 expresses the SPI-2 genes in similar or even moderately higher levels than *S*. Typhimurium SL1344 (S3B Fig). We concluded from these results that the impaired replication of this strain is not the result of insufficient expression of SPI-2 genes that are normally induced intracellularly, but is likely due to a different reason; possibly inactivating mutations in several T3SS-2 effector genes and pSLT absence (see Discussion).

### The sparrow MpSTM infects and causes severe inflammation in an avian host

**Fig 4:**
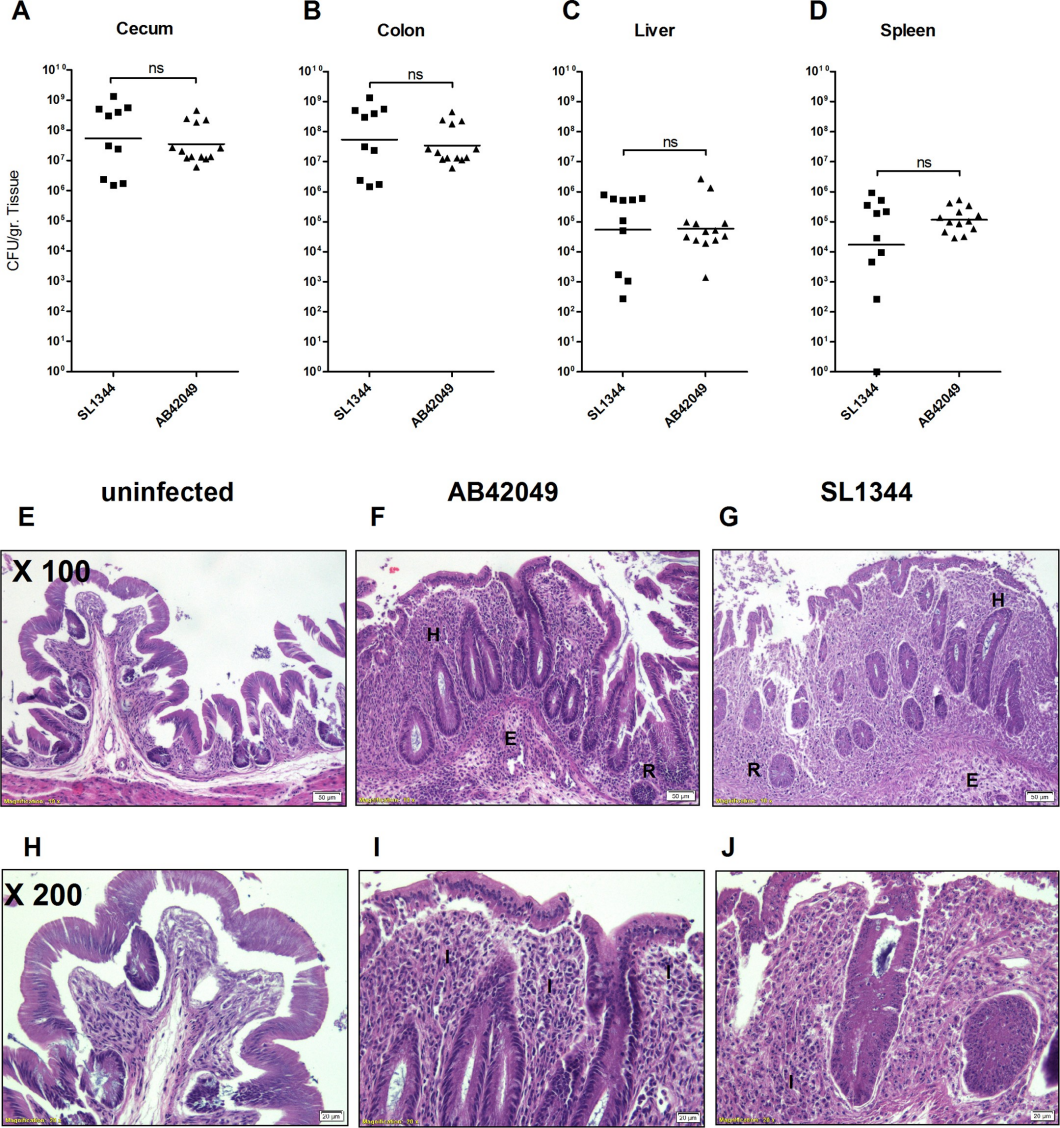
The *S*. Typhimurium sparrow-associated strain readily infect chicks and induce severe pathology. (**A-D**) Groups of one-day old SPF chicks were intracrop infected with 5–8×10^6^ CFU of *S*. Typhimurium SL1344 or AB42049 harboring ampicillin resistance. At day-four p.i., intestinal (cecum and colon) and systemic (liver and spleen) organs were aseptically isolated, weighted and homogenized in saline. Serial dilutions were plated on selective XLD plates to determine the bacterial loads in the cecum (**A**), colon (**B**), liver (**C**) and spleen (**D**). The experiment was conducted twice and combined data from the two independent experiments are shown. Each dot represents data from one bird and horizontal lines show the geometrical mean of bacterial load, per gram of tissue. T-test was used to determine statistical significance (ns, not significant). (**E-F**) Cecal tissues from uninfected control and from infected chicks were fixed in formalin for 24 h and embedded in paraffin. Sections were stained with Hematoxylin-eosin and imaged for pathology assessment. (**E-G**) × 100 magnifications are shown, bar = 50 μM. The following signs of pathology is indicated: E, edema; H, hyperplasia; R, regenerative crypt changes; I, infiltration of immune cells. (**H-J**) × 200 magnifications are shown, bar = 20 μM.

Subsequently, we characterized the pathogenicity of the sparrow MpSTM in an avian host. One-day-old SPF White Leghorns chicks (Charles River) were infected with 5–8×10^6^ CFU of *S*. Typhimurium AB42049 or SL1344. At day-four post infection, similar levels of both strains were recovered from the intestines and systemic sites of the chicks (Fig 4A–4D). H&E staining of cecal sections from chicks infected with SL1344 and AB42049 showed similar pathology with clear signs of necrosis in the lamina propria, mucosa and in some part of the epithelial layers, villus effacement and disruption of intestinal crypts, hyperplasia, and edema in parts of the submucosa. Severe inflammation was evidenced by massive infiltration of heterophils, lymphocytes and macrophages (Fig 4E–4J).

**Fig 5:**
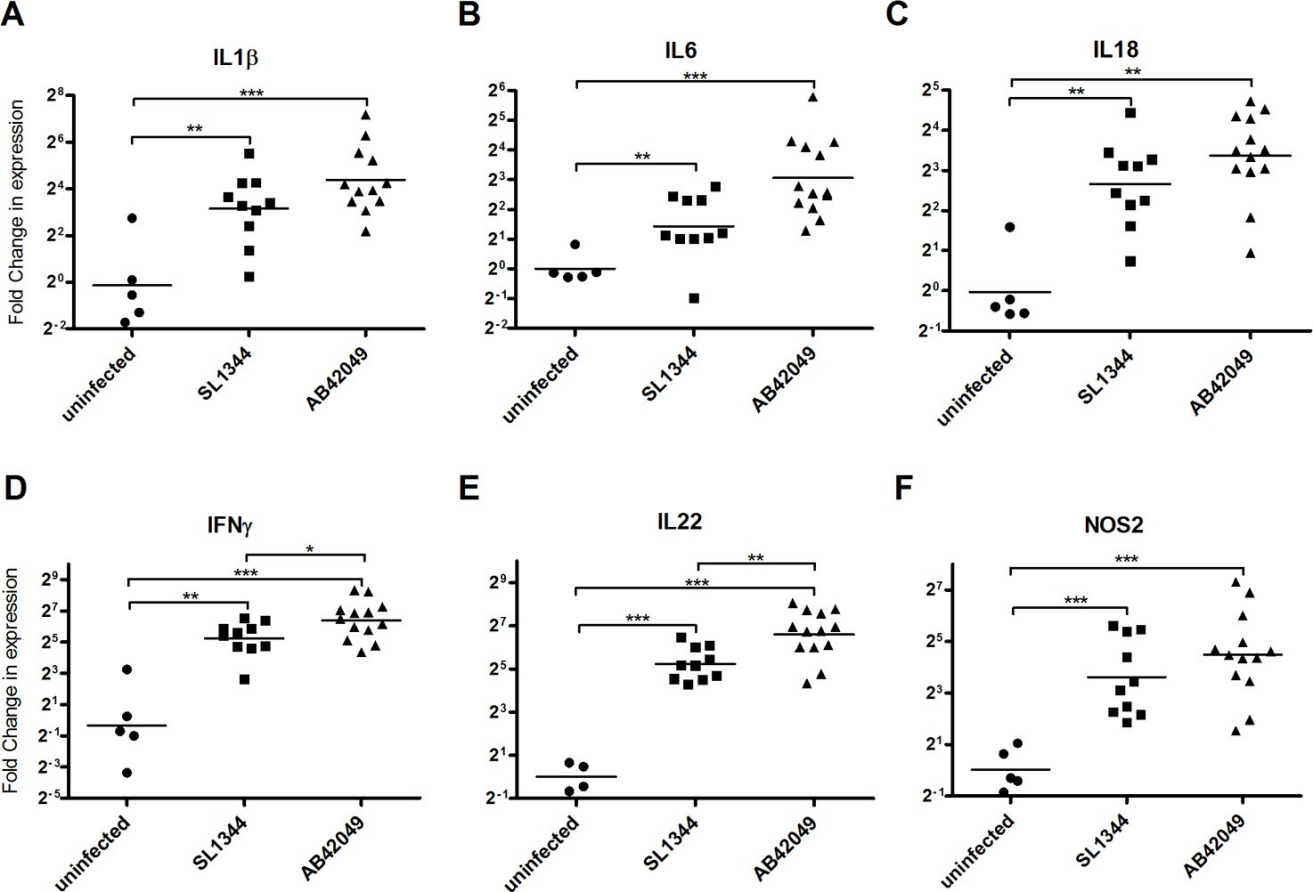
The *S*. Typhimurium sparrow-adapted strain causes severe inflammation in an avian host. RNA was extracted from cecal tissues of uninfected chicks and from birds that were infected with *S*. Typhimurium SL1344 or the sparrow isolate AB42049 four days p.i. The relative expression of the proinflammatory cytokine genes *IL1B* (**A**), *IL6* (**B**), *IL18* (**C**), *IFNG* (**D**), *IL22* (**E**), and *NOS2* (**F**) in the infected chicks is shown relative to their expression in uninfected birds. The expression was normalized to the housekeeping gene *Gapdh*. Each dot represents the qRT-PCR data from one bird and the geometrical mean is shown by the horizontal line. *, *P*<0.05; **, *P*<0.01; ***, *P*<0.001.

Analysis of proinflammatory cytokine expression in cecum of infected chicks that was examined by qRT-PCR further showed induced expression of *IL1B*, *IL6*, *IL18*, *IFNG*, *IL22* and *NOS2* genes, in birds that were infected with AB42049 or SL1344 relative to uninfected animals (Fig 5). Moreover, the expression levels of *IFNG* and *IL22* was moderately, but significantly higher in the chicks infected with AB42049 compared to chicks infected with SL1344.

Collectively, these results indicate that the sparrow-associated strain can infect the avian host, cause acute intestinal inflammation and colonization at systemic sites at similar levels as *S*. Typhimurium SL13444.

### The sparrow MpSTM presents impaired pathogenicity in the mouse

**Fig 6:**
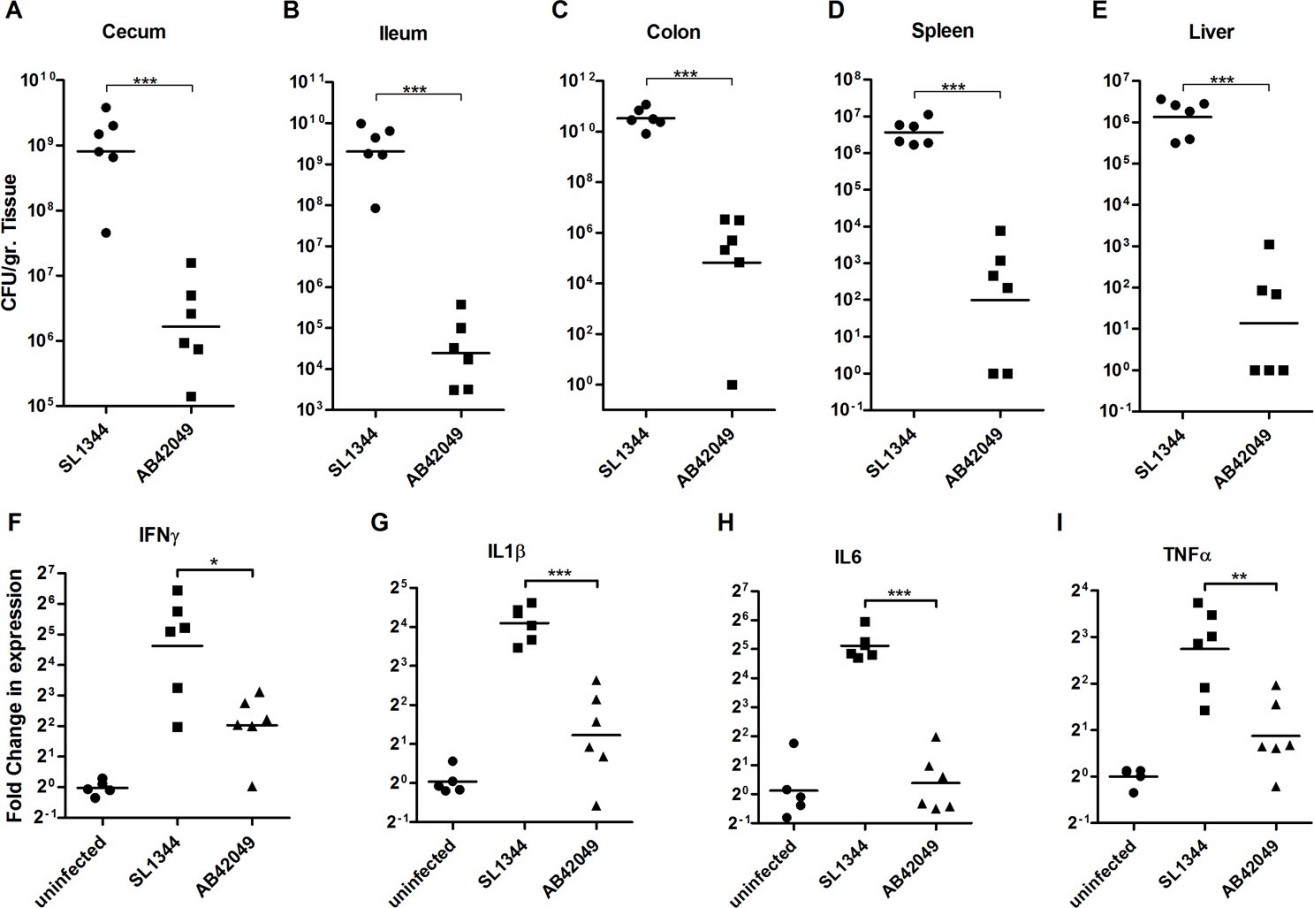
The *S*. Typhimurium sparrow-associated strain has impaired virulence in the mouse. (**A-E**) Groups of C57/BL6 mice were orally infected with ~1×10^6^ CFU of *S*. Typhimurium SL1344 and the sparrow-associated strain AB42049, harboring ampicillin resistance. At day-four p.i., tissues were collected and homogenized in saline. Serial dilutions of the homogenates were plated on selective XLD agar plates. Bacterial loads per gram of tissue in the cecum (**A**), ileum (**B**), colon (**C**), spleen (**D**) and liver (**E**) are shown. Each dot represents the count from a single mouse and the geometric mean is indicated by the horizontal line. (**F-I**) RNA was extracted from liver tissues of uninfected mice and from mice that were infected with *S*. Typhimurium SL1344 or the sparrow isolate AB42049 4-days p.i. The relative expression of the proinflammatory cytokine genes *Ifng* (**F**), *Il1b* (**G**), *Il6* (**H**), and *Tnfa* (**I**) in the infected mice is shown relative to their expression in uninfected animals. The expression was normalized to the housekeeping gene *Gapdh*. Each dot represents the qRT-PCR data from one mouse and the geometrical mean is shown by the horizontal line. A Student t-Test was used to determine statistical significance *, *P*<0.05; **, *P*<0.01; ***, *P*<0.001.

To test the adaptation of the sparrow MpSTM strain to a mammalian host, its pathogenicity was studied in the mouse model. Streptomycin pretreated mice were infected with ~1×10^6^ CFU of *S*. Typhimurium AB42049 or SL1344 by oral gavage. Strikingly, in contrast to the similar colonization that was found in chicks, in the mouse, the colonization of *S*. Typhimurium AB42049 was three to six logs lower than SL1344 (Fig 6A–6E). These differences were especially pronounced in systemic sites, where low colonization of AB42049 was observed in the liver (median 35 CFU/ gr of tissue) and spleen (median 340 CFU/ gr of tissue). To characterize these differences further, we determined the expression levels of proinflammatory cytokines in the liver of infected mice. In agreement with the differences in bacterial burden, much lower mRNA expression of *Ifng*, *Il1b*, *Il6* and *Tnfa*, was measured in mice infected with AB42049 compared to SL1344 (Fig 6F–6I), indicating that the sparrow-associated strain induces much lower inflammatory response in mice than the reference *S*. Typhimurium strain.

**Fig 7:**
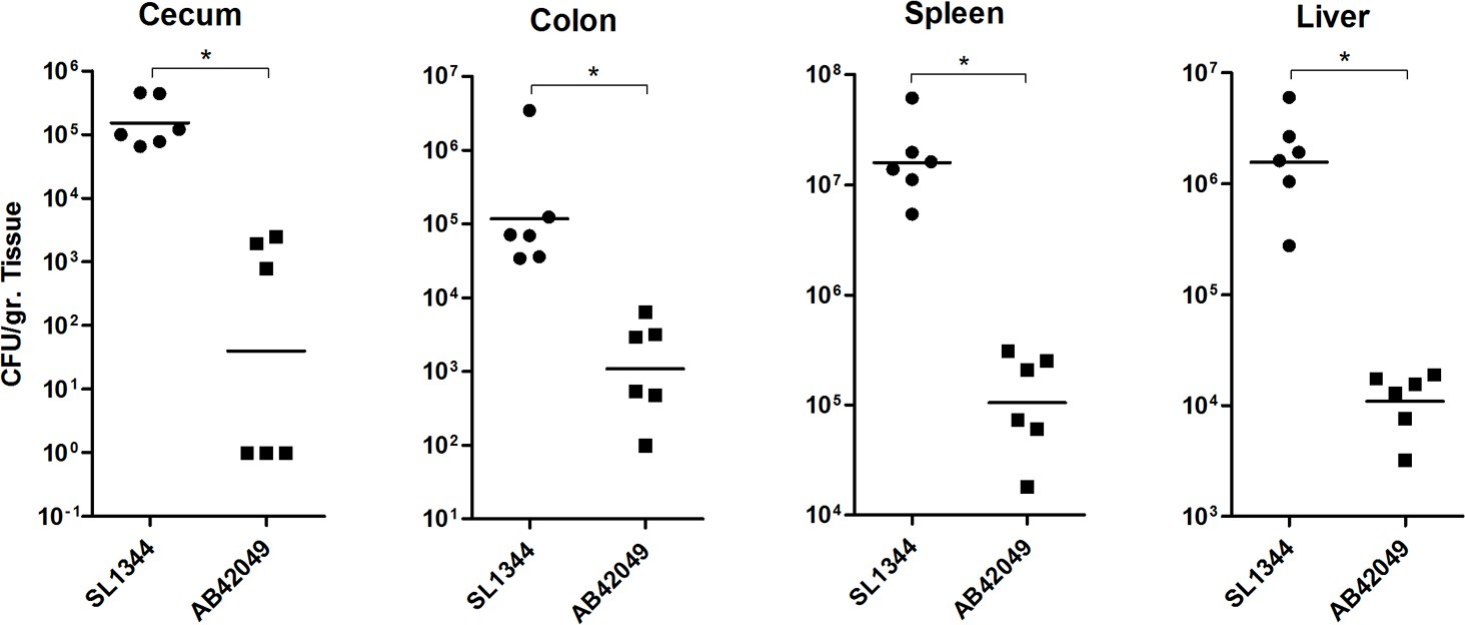
Intraperitoneal injection retains the impaired virulence of AB42049 in mice. Two groups of C57/BL6 mice were infected i.p. with ∼3×10^4^ CFU of *S*. Typhimurium SL1344 and the sparrow-associated strain AB42049, harboring ampicillin resistance. At day-three p.i., organs were collected and homogenized in saline. Serial dilutions of the homogenates were plated on selective XLD agar plates. Bacterial loads per gram of tissue in the cecum (**A**), colon (**B**), spleen (**C**) and liver (**D**) are shown. Each dot represents the count from a single mouse and the geometric mean is indicated by the horizontal line. A Student t-Test was used to determine statistical significance, *, *P*<0.05.

To test whether the impaired virulence of AB42049 in mice is dependent on the infection route, we infected C57/BL6 mice intraperitoneally (i.p.) with ∼3×10^4^ CFU of *S*. Typhimurium AB42049 or SL1344 strains. Bacterial loads at the spleen, liver, colon and cecum were determined at day three p.i. (Fig 7). Interestingly, subsequent i.p. infection that bypasses the passage of *Salmonella* through the intestinal system, the differences in bacterial loads between SL1344 and AB42049 were reduced, but still the colonization of the sparrow isolate in the mouse was lower by 2–3 orders of magnitude compared to SL1344.

Collectively, these results demonstrate that while *S*. Typhimurium SL1344 and the sparrow MpSTM are capable of causing comparable and sever disease in an avian host, AB42049 colonizes to much lower extent the mouse intestines and cause much milder systemic disease compared to SL1344, its closely related strain.

### Association of pseudogenes found in AB42049 with *S*. Typhimurium human and avian isolates

**Fig 8:**
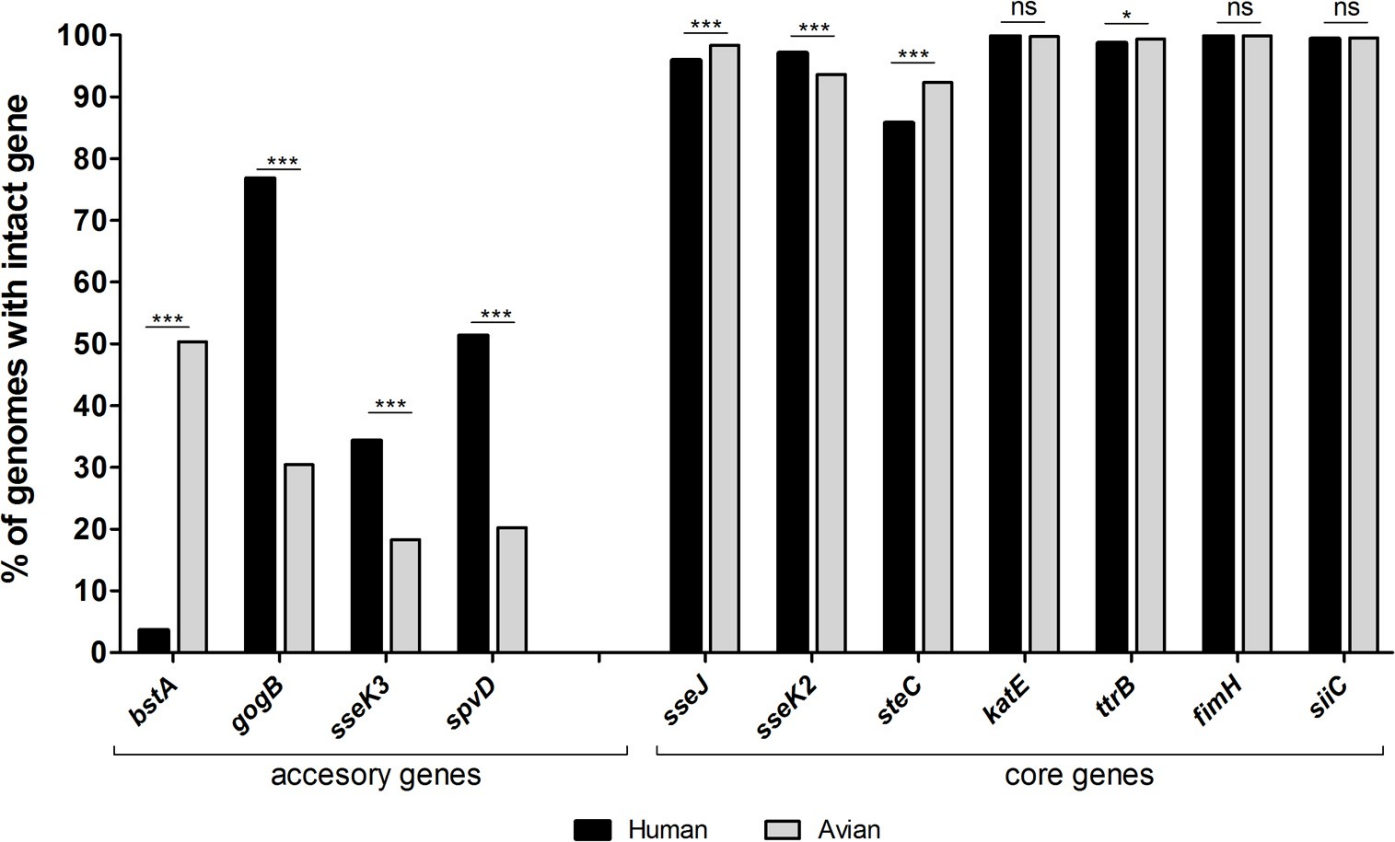
Distribution of pseudogenes found in AB42049 among human and avian *S*. Typhimurium isolates. The frequency of accessory (*bstA*, *gogB*, *sseK3* and *spvD*) and core (*sseJ*, *sseK2*, *steC*, *katE*, *ttrB*, *fimH*, and *siiC*) genes was determined among 2972 and 3134 genome assemblies of *S*. Typhimurium isolates from avian and human origin, respectively. The frequency of each gene is shown as the percentage of genomes that harbor an intact gene from the entire population group. The presence and the integrity of each gene were examined using tblastn against the corresponding protein sequence of *S*. Typhimurium SL1344 or ST313. Translated genes from subject genomes that presented ≥ 90% identity with ≥ 90% alignment with the protein query were considered intact. Statistical difference between the occurrences of each gene among human and avian isolates was determined by the z score test for two population proportions. *, *P*<0.05; ***, *P*<0.001; ns, not significant.

The absence of pSLT and accumulation of multiple pseudogenes in the T3SS-2 regulon of AB42049, its impaired intracellular replication and its reduced ability to cause a systemic infection in the mouse, have led us to hypothesize that specific genetic makeup may contribute to the adaptation of this strain to the avian host. To test this hypothesis, we analyzed the presence and integrity of the genes *gogB*, *sseK2*, *sseK3*, *sseJ*, *steC*, *katE*, *ttrB*, *fimH*, *siiC*, *spvD* (encoded on pSLT), and *btsA* (encoded by the BTP1 prophage) among 3134 human and 2972 avian isolates of *S*. Typhimurium, registered in Enterobase. Interestingly, we found significant differences in the distribution of four accessory genes, including much higher presence of intact *gogB*, *spvD*, and *sseK3* genes in genomes of human isolates vs. avian isolates (76.8, 51.3 and 34.3% vs. 30.4, 20.2 and 18.3%, respectively; Fig 8). In contrast, we identified much higher prevalence of intact *bstA* phage gene among avian isolates (50.3%) compared to human isolates (3.6%). Statistically significance differences were also found in the prevalence of the core genes *sseJ*, *sseK2*, *SteC* and *ttrB*, however in these cases, the difference is very low (ranges between 0.6% and 6%) and its biological significance is unclear.

These results indicate that the association of intact presence of *bstA* and the absence or inactivation of *spvD*, *sseK3* and *gogB* are much higher among poultry isolates of *S*. Typhimurium and that collectively this specific genetic pattern may contribute in a multifactorial manner to the adaptation of the sparrow MpSTM strain to the avian host.

## Discussion

During *Salmonella* evolution, many different ecological niches have effectively occupied by this highly diverse bacterial pathogen. While many *S*. *enterica* serovars successfully maintained their ability to infect and colonize in a wide-array of host species, a few serovars or clonal lineages have evolved to colonize and cause a disease in one or a small group of hosts. These pathogens have emerged relatively recently from generalist ancestors by restricting their host range and modifying their pathogenicity [38]. The evolutionary dynamic of *Salmonella* host-adaptation is still not fully understood, but is appreciated as multifactorial, complex, and assumed to involve different cellular and metabolic pathways [39,40].

Nonetheless, a recurring theme in narrowing host range from a generalist to a specialist pathogen is known as genome degradation or genomic decay. This process involves gene loss or gene inactivation through pseudogene formation of metabolic and virulence functions that are presumably no longer needed in the specific adapted host. Such loss of function mutations allows increasing the adaptation to a particular host, on the expense of reducing the fitness in the environment and in other ecological niches [39]. Evidences for genomic decay are prominent across all host-adapted *Salmonella* serovars. For example, while the genome of the generalist *S*. Typhimurium (strain LT2) contains only 25 pseudogenes [41], much higher rate of pseudogenes have been determined in the genomes of host-adaptive pathotypes, including *S*. Typhi [42], *S*. Paratyphi A [43], *S*. Paratyphi C [44], *S*. Choleraesuis [45], *S*. Dublin [46], *S*. Gallinarum [41], and, *S*. Typhimurium DT2 [38].

Similarly, the presence of multiple pseudogenes in the genome of *S*. Typhimurium ST313, has led to the hypothesis that this lineage is in a process of adaptive evolution [47,48]. Comparable to these host-adapted strains, the sparrow AB42049 isolate characterized here harbors 67 chromosomally encoded pseudogenes and a complete loss of the virulence pSLT plasmid, typical to *S*. Typhimurium isolates [25]. Interestingly, many of the pseudogenes found are known to play a role in host cells adhesion (*bigA*, *ratB*, *siic*, *siiE*, *lpfD*, and *fimH*), anaerobic respiration (*ttrB*), oxidative stress tolerance (*katE*) and intracellular survival and systemic infection (*gogB*, *sseK2*, *sseK3*, *sseJ*, *steC* and pSLT). Similar pseudogenes profile was recently described in the genetically similar *S*. Typhimurium DT56 passerine isolate (strain SO7676-03) isolated at 2005 at northern England [16,24], with only 141 variations (most of which are SNPs) distinguishing these two strains.

Alteration in the ability of a *Salmonella* to adhere to host cells is one of the most characterized mechanisms affecting *Salmonella* host-specificity. For example, the *misL* and *shdA* genes encoding nonfimbrial adhesins play a role in *S*. Typhimurium intestinal colonization [49,50]. Pseudogene formation leading to *shdA* inactivation has been demonstrated in *S*. Typhi [42], *S*. Paratyphi [43], *S*. Paratyphi C [44], *S*. Dublin [46], and *S*. Gallinarum [41], and psuedogene formation in *misL* was shown in *S*. Typhi [42] and is associated with the human adaptation of these serovars. Similarly, genome degradation in chaperon-usher fimbrial genes was described in 7 out of 11 fimbrial operons in *S*. Typhi [51], in 3 of 12 fimbrial operons in *S*. Paratyphi A [43] and in 8 of 12 fimbrial operons in *S*. Gallinarum [41]. Based on that, pseudogene formation in the nonfimbrial adhesin genes *bigA*, *ratB*, and *siiE*, together with inactivation of the chaperon-usher adhesin genes *lpfD*, and *fimH*, identified in the genome of the new sparrow MpSTM may contribute to the unique host-tropism of this strain.

Metabolic pathways that allow *Salmonella* better energy consumption and superior growth than the gut microbiota of certain hosts may also adjust host-specificity. While the broad-host-specificity serovar *S*. Typhimurium utilizes tetrathionate respiration encoded by the intact *ttrRS/ttrBCA* gene cluster to gain metabolic advantage over the gut microbiota [28], pseudogenes in this pathway were identified in the host-specific *S*. Typhi (*ttrS*) [42] and *S*. Gallinarum (*ttrB*, *ttrC*) [41]. Thus, inactivation of *ttrB*, in the genome of the sparrow MpSTM, suggests possible change in the interaction with the microbiota that may help to restrict its intestinal colonization in mammalian hosts.

Notwithstanding, probably the most intriguing genome decay in AB42049 is multiple pseudogenes in T3SS-2 effectors and the loss of pSLT. No less than five effector genes (*gogB*. *sseK2*, *sseK3*. *sseJ*, and *steE*) and the stationary-phase catalase, *katE* are inactivated in this strain. Since these genes are involved in the systemic and intracellular phase of *Salmonella* infection, required for SCV formation (*sseJ* and *steC*), suppression of innate immune signaling pathways (*gogB*, *sseK2* and *sseK3*) [10], and persistence inside fibroblasts (*sseJ*) [11] we expect that these mutations play a critical role in the pathoadaptation of this strain. Additionally, the absence of pSLT, encoding the *spv* operon, previously shown to contribute to *Salmonella* intracellular replication [52,53] and virulence in mice [54] is presumed to further contribute to the host specificity of this strain. In agreement with this hypothesis, we showed that AB42049 presents impaired ability to replicate within non-phagocytic host cells, reduced localization to the SCVs and attenuated virulence in the mouse, in comparison with the reference *S*. Typhimurium strain. Previous studies have shown that even a single deletion of either *sseJ* [55], *steC* [56], or *spvB* [52] significantly affects *S*. Typhimurium virulence in the mouse. Moreover, the occurrence of gene inactivation or the complete absence of *gogB*, *spvD*, and *sseK3* in poultry isolates of *S*. Typhimurium was much higher than in human isolates. Taken together, these accumulative observations suggest that the above genes are required for pathogenicity of *S*. Typhimurium in humans or mammals, but may be dispensable in the avian host.

Additional genomic signature associated with *Salmonella* host-specificity is the acquisition of prophages. For example, *S*. Typhimurium ST313 harbors five full-length prophages including the ST313-specific, BTP1 phage [26]. Unexpectedly, AB42049 was also found to harbor an intact BTP1 phage. One of the genes encoded by BTP1 called *bstA* (st313-td) was recently shown to be widely present in isolates of the bovine-adapted serovar, *S*. Dublin. There, *bstA* acts as an anti-virulence gene, as *S*. Dublin that was deleted from *bstA* was more virulent than the wild-type strain in the mouse model [57]. Another common features between *S*. Typhimurium ST313 and the sparrow-adapted STM are the inactivation of the gene *katE*, and the colonization factor encoded gene *ratB* [58]. In the case of *S*. Typhimurium ST313, it was speculated that due to its adapted systemic lifestyle, it no longer requires KatE for transmission and/or survival in the environment [58]. In agreement with this notion, we showed that like *S*. Gallinarum, the sparrow-associated strain is unable to form biofilm, and is sensitive to hydrogen peroxide in comparison to *S*. Typhimurium SL1344. These data suggest that the presence of BTP1, in conjunction with the inactivation of additional virulence and metabolic genes, may contribute to the pathoadaptation and possibly to the attenuated virulence of AB42049 in the mouse host, while demonstrating convergent evolution with the distinct pathovariant, *S*. Typhimurium ST313.

Nevertheless, we expect that this complex phenotype is multifactorial and does not result from the presence or absence of one gene only, as the complementation of *katE* or *sseJ* alone did not rescue the impaired intracellular replication of AB42049 in DF-1 cells (S4 Fig). In addition, infecting mice i.p. with the AB42049 strain reduced the magnitude of difference in bacterial loads compared to SL1344, but did not fully overcome the impaired virulence that was observed following oral infection. These results suggest that the overall compromised pathogenicity presented by AB42049 in mice is probably shaped by independent process occur during both the intestinal and the systemic phases of the infection.

In summary, we report the isolation and characterization of a MpSTM strain that was isolated from wild sparrows in Israel. This strain presented normal invasion phenotype into non-phagocytic cells, but impaired intracellular replication. Concurring, this strain exhibited attenuated virulence in mice, but was fully virulent in the chick model, indicating pathoadaptation to the avian host. Genome analysis demonstrated the presence of the BTP1 prophage, complete loss of the virulence plasmid, pSLT and genome degradation in multiple adhesion factors, tetrathionate respiration and several T3SS-2 effectors genes. To the best of our knowledge, *Salmonella* host-adaptation has never been linked to genome degradation in T3SS-2 and pSLT loci, but it is highly likely that this pattern of genome decay significantly restricts the virulence of this strain in the mouse, while permitting full pathogenicity in the avian host. Furthermore, the fact that this strain was isolated from four independent birds in one geographic location, suggests that it is highly infectious and presents high transmission capability between wild birds in nature. Overall, our findings demonstrate the unique phenotypes and genetic makeup of an overlooked pathotype of monophasic *S*. Typhimurium and present distinct pathways expected to facilitate pathoadaptation to passerine birds.

## Materials and methods

### Ethics statement

Mouse experiments were conducted according to the ethical requirements of the Animal Care Committee of the Sheba Medical Center (Approval # 1182/18) and in line with the national guidelines. *Salmonella* infection of chicks was carried out according to the ethical requirements of the Animal Care Committee of the Sheba Medical Center (Approval numbers 1059/16) and in line with the guidelines of the National Council for Animal Experimentation.

### *Salmonella* spp. isolation and bacterial strains

Bacterial strains included in this study are listed in S3 Table. Wild birds that were caught using a mist net by licensed ringers were sampled by noninvasive cloacal swabs. Samples were resuspended in tryptic soy broth medium (BD Difco) and grown for overnight at 37°C. Cultures were diluted 1:100 into fresh Rappaport Vassiliadis R10, *Salmonella* enrichment broth (BD Difco), incubated at 42°C for 16 h and streaked on Xylose Lysine Deoxycholate (XLD) agar plates. *Salmonella* suspected colonies (colored in black) were tested by PCR using *invA*-specific primers (S4 Table). PCR-confirmed *Salmonella* isolates were serotyped at the *Salmonella* National Reference Center using specific O and H antisera according to the Kauffmann-White scheme [1].

### Molecular biology

Primers used in this study are listed in S4 Table. STM SL1344 *katE* null mutant strain was constructed using the λ-red-recombination system [59]. PCR was applied to produce an amplimer containing kanamycin resistance gene using the primers katE_P1 and katE_P2 that was integrated into the genome of SL1344 and replaced the *katE* locus. The resistant cassette was then excised from the genome using a helper plasmid pCP20 encoding the FLP recombinase. The resulted null non-polar deletion of *katE* was verified by Sanger sequencing of a PCR product amplified using the primers katE_5’_flank and katE_3’_flank.

To complement the expression of *katE* and *sseJ* in AB42049, *katE* was amplified from STM SL1344 using the primers OA003F and OA003R. The resulted product was digested with XhoI and XbaI and cloned into pWSK29. *sseJ* was PCR amplified using the primers OA004F and OA004R digested with SacI and XbaI and cloned into pWSK29 as well. Both constructs were introduced into electrocompatent AB42049 cells.

### Pulsed-field gel electrophoresis (PFGE)

PFGE analysis was carried out according to the standardized *Salmonella* protocol defined by CDC PulseNet and as was previously detailed [60], using *S*. Braenderup H9812 strain as a molecular standard.

### Motility assay

The motility phenotype was conducted as previously reported [61].

### Biofilm formation

*Salmonella* cultures were grown overnight in LB-Lennox broth and subcultured 1:100 into fresh LB medium without NaCl (10 g/L peptone, 5 g/L yeast extract) and 150 μl of the culture were added into cell-culture-treated 96-well microplates. The plates were statically incubated at 28°C for 96 h. Planktonic cells were discarded and the attached biofilm was fixed for 2 h at 60°C. Fixed biofilm was stained with 150 μl of 0.1% Crystal Violet for 10 min at room temperature and washed with PBS. The dye bound to the biofilm was resuspended in 150 μl of 33% acetic acid and measured at 560 nm.

### Hydrogen peroxide resistance

*Salmonella* cultures were grown overnight in LB medium, washed with saline and resuspended in 0, 50 and 100 mM of H_2_O_2_. The suspensions were incubated at 37°C for 20 minutes and serially diluted in saline. Spots of 10 μl from each dilution were plated on LB-agar plates at 37°C and imaged using Fusion SOLO X system (VILBER).

### Reverse transcription real-time PCR

RNA was extracted from *S*. Typhimurium cultures grown under different conditions using the RNA protect bacterial reagent and the RNeasy mini kit (QIAGEN) according to the manufacturer’s instructions, including an on-column DNase I digest. Purified RNA was retreated with an RNase-free DNase I followed by ethanol precipitation. 200 ng of DNase I-treated RNA was subjected to cDNA synthesis using the qScript cDNA synthesis kit (Quantabio). Real-time PCR and data analysis were performed as previously described [62] on a StepOnePlus Real-Time PCR System (Applied Biosystems). The *rpoD* and 16S rRNA gene were used as the endogenous normalization controls. Fold-differences in gene transcription were calculated as 2^-ΔΔC_t_^.

### Host cell invasion and replication tissue cultures

Human epithelial HeLa (ATCC CCL-2) and chicken fibroblasts DF-1 (ATCC CRL12203) cells were purchased from the American Type Culture Collection and were cultured in a high-glucose (4.5 g/liter) DMEM supplemented with 10% FBS, 1 mM pyruvate and 2 mM L-glutamine at 37°C in a humidified atmosphere with 5% CO_2_. Cells were seeded at 5×10^4^ cells/ml in a 24-well tissue culture dish 18 h prior to bacterial infection and infected at multiplicity of infection (MOI) of 45 (bacteria per cell). Infection experiments were carried out using the gentamicin protection assay as previously described [63].

### Flow cytometry

DF-1 chicken fibroblasts were seeded at 4×10^5^ cells/ml in a 6-well tissue culture dish 18 h prior to bacterial infection and infected at MOI of 25. Cells were infected with *S*. Typhimurium SL1344 or AB42049 carrying the P_*ssaG*_::sfGFP or P_*uhpT*_::sfGFP plasmids. Following infection, the cells were centrifuged at 150 g to synchronize the infection and incubated at 37°C in a humidified atmosphere with 5% CO2. 30 minutes p.i. the cells were washed with PBS and DMEM supplemented with 100 μg/ ml gentamicin was added. Following incubation of 90 min, the medium was replaced with fresh DMEM containing 10 μg/ ml gentamicin and cells were incubated for additional 6 h. At 8 h p.i, infected DF-1 cells were collected by trypsinization, fixed with 4% paraformaldehyde for 20 min at room temperature and washed with PBS containing 5 mM EDTA. The presence of *Salmonella* in the cytoplasm and SCV was analyzed with the CytoFlex LX (Beckman Coulter, Inc.) system at flow rate of 60 μl/min. 561 nm laser was used to collect 1×10^4^ dsRED expressing cells, while the 488 nm laser was used to analyze the number of cells expressing GFP. The FACS results were analyzed using the Kaluza software (Beckman Coulter, Inc.).

### Chick infection model

*Salmonella* infection of chicks was carried out as previously reported [64], with some modifications. Briefly, SPF eggs of White Leghorns chicks (Charles River) were incubated for 21 days at 37.4°C in SPF isolators. One day after hatching, the chicks were orally (intra crop) infected with 5–8×10^6^ CFU of *Salmonella* harboring ampicillin resistance that were grown for 16 h in LB at 37°C. Four days p.i. the chicks were sacrificed and the systemic and GI organs were aseptically collected on ice and homogenized in saline. Serial dilutions were plated on XLD agar plates supplemented with ampicillin for bacterial counting.

### Mouse infection model

Streptomycin (20 mg per mouse) was given by oral gavage 24 h prior to the infection to 7 week-old Female C57/BL6 mice (Envigo, Israel). *Salmonella* Typhimurium strains harboring pWSK29 were grown in selective LB broth for 16 h and diluted in 0.2 ml saline. Mice were orally infected using gavage needle with ~1×10^6^ CFU of each strain. For i.p. infection, two groups of six mice each were infected with ∼3×10^4^ CFU of STM SL1344 or AB42049 harboring pWSK29. At day-three or -four post-infection, following i.p and oral infection, respectively mice were euthanized and tissues were collected on ice and homogenized. Serial dilutions of the homogenates were plated on XLD agar plates under ampicillin selection, and counted to calculate bacterial tissue loads.

### Histology

Tissues were fixed in 10% neutral buffered formalin for 24 h and then embedded in paraffin. Sections (5 μm) were stained with Hematoxylin and Eosin (H&E). Pictures were taken using Olympus BX60 Microscope at objective magnification of ×10 and imaged with Olympus DP73 digital camera.

### Cytokine expression *in vivo*

Cecal and liver tissues that were isolated from infected mice and chicks were immediately preserved in RNAlater Stabilization Reagent (QIAGEN) and RNA was extracted using RNeasy Plus Mini Kit (QIAGEN). Extracted RNA was reverse transcribed into cDNA using the qScript cDNA synthesis kit (Quantabio). Quantitative real-time PCR (qPCR) was performed with Fast SYBR Green Master Mix (Applied Biosystems) and the gene-specific primers (S4 Table) on a StepOnePlus Real-Time PCR System. Data were normalized to the house-keeping gene *Gapdh* and fold change was calculated as 2^-ΔΔC_t_^.

### Whole genome sequencing

DNA from *S*. Typhimurium strain AB42049 was isolated from an overnight culture grown in LB using the GenElute Bacterial Genomic DNA Kit (Sigma-Aldrich). Whole genome sequencing that was performed at the Technion Genomic Center (Haifa, Israel) has generated 7×10^6^ paired-ends 250 bp reads by an Illumina MiSeq platform (Illumina, Inc.) and 234,215 long reads using a MinION sequencer (Oxford Nanopore Technologies). The long reads (mean: 12,140 bp; N50: 29,623 bp) were used for *de novo* assembly using the Trycycler (v 0.3.3) pipeline, according the developer’s instructions. The assembled genome was then polished with the short reads to correct possible sequencing errors, resulting in sequencing depth of 760×. The complete gap-free genome of isolate AB42049 was deposited at NCBI under accession number CP064919 (BioProject number: PRJNA671808).

Isolates AB42052, AB42086, and AB42142 were sequenced on MinION sequencer (Oxford Nanopore Technologies). Statistical analysis and quality control of the MinION reads were done using NanoPlot (v 1.33.1). The number of reads generated for AB42052, AB42086 and AB42142 was 253,417, 264,623, and 251,790 respectively. The mean length of the reads was 12,505, 11,991 and 12,860 bp and the N50 was 30,089, 29,285 and 30,282 for AB42052, AB42086 and AB42142, respectively. The generated reads were used for *de novo* assembly using Trycycler pipline (v 0.3.3), resulting in assembled genomes with sequencing depth of 661, 255, and 250× for AB42052, AB42086 and AB42142, respectively. These genomes were deposited under accession numbers CP064917 (AB42086), CP064918 (AB42052), and CP064916 (AB42142).

### Bioinformatic analyses and tools

The assembled genome of isolate AB42049 was annotated with DFAST [65] using the published genomes of STM SL1344 (NC_016810) and SO7676-03 (PRJEB34599) as references. DFAST functional annotation includes prediction of pseudogenes by re-aligning the CDS and its flanking region to its orthologous protein by implementing LAST [66] with a subject coverage cutoff of 85%. The pseudogenes were then identified by filtering the gff file with ‘pseudogene’, ‘stop codon’ or ‘frameshift’ terms that were manually curated. Sequences alignment was conducted using Mauve [67] and compared by BRIG [68]. Phages and their integration sites were identified using PHASTER [69]. BTP1 region comparison between AB42049 and *S*. Typhimurium ST313 (D23580) was carried out using the genome comparison visualizer, EasyFig [70].

To create the phylogenetic tree of isolate AB42049, 38 genome assemblies of MpSTM isolated from avian or poultry origin were downloaded from Enterobase [71] and used to create a phylogenetic tree together with the genomes of *S*. Typhimurium strains AB42049, SO7676-03 (NZ_LR862421), ST313 (NC_016854) and SL1344 (NC_016810.1) that was used as the tree root. The phylogenetic tree was constructed using the PhaME software [72]. All the genome assemblies used to build this tree are listed in S5 Table.

Variant calling between the genomes of SO7676-03 and AB42049 was done by comparing the published genome of SO7676-03 (PRJEB34599) against the short reads of MpSTM AB42049 with Snippy v4.6.0 (https://github.com/tseemann/snippy).

To determine the occurrence of genes of interest in the genomes of *S*. Typhimurium isolates, all of the 2972 genome assemblies of avian isolates (N = 2972) available from Enterobase (as for Nov. 2020) were selected for the analysis. For human isolates, we have selected and downloaded from Enterobase the available 3134 genome assemblies of *S*. Typhimurium clinical isolates with the highest genome coverage (869 to 124×). The genome assemblies (listed in S6 Table) were compared using tblastn against the appropriate reference protein sequences of *S*. Typhimurium SL1344 (NC_016810.1) or ST313 (NC_016854). Translated genes that presented ≥ 90% identity over ≥ 90% of the alignment length were considered intact.

## Supporting information

S1 Fig The *S*. Typhimurium sparrow adapted strain harbors the ST313 BTP1-associated phage. Analysis of the sparrow-associated strain AB42049 genome by PHASTER identified the presence of the *S*. Typhimurium ST 313 prophage BTP1. Pairwise alignment between the BTP1 region of *S*. Typhimurium ST313 str. D23580 (NC_016854; position 366797–410321) and the corresponding region of AB42049 (position 365947–409906) is shown. Sequence homology is illustrated by the shades of grey.
(TIF)

S2 Fig Genome degradation of T3SS-2 genes in the *S*. Typhimurium sparrow-associated strain. Amino acids alignment of the T3SS-2 effector proteins SseJ (**A**), SteC (**B**), SseK2 (**C**), SseK3 (**D**), and GogB (**D**) and the KatE catalase (**F**) between their wildtype sequence in *S*. Typhimurium SL1344 and their inactivated sequence in AB42049 is shown. All pseudogenes identified in the AB42049 assembly were confirmed by PCR amplification and Sanger sequencing of the resulted product.
(PDF)

S3 Fig SPI-2 genes are expressed normally in the *S*. Typhimurium sparrow–associated strain. (**A**) *S*. Typhimurium SL1344 and AB42049 strains were grown to the late logarithmic phase and used to infect DF-1 cells at MOI of 25. 8 h p.i. RNA was extracted from the infected cells and from the LB grown cultures. qRT-PCR was used to determine the fold change in expression of *ssrB*, *ssaR*, *ssaV*, *sseG*, *sseD*, and *sifA* in intracellular *Salmonella* vs. their expression in LB grown cultures. The house keeping genes *rpoD* and 16S rRNA were used for normalization of target genes. The values represent the fold change of the intracellular expression compared to the expression in LB culture. (**B**) The fold change in the expression of *ssrB*, *ssaC*, *ssaV*, *sseA*, *sseD*, *sseG*, *sifA* and *SifB* was determined for intracellular AB42049 relative to the expression of these genes in intracellular *S*. Typhimurium SL1344 using qRT-PCR. The indicated values show the mean of three repeats and the SEM is represented by the error bars.
(TIF)

S4 Fig Intracellular replication of AB42049 and its complemented strains in DF-1 cells. Intracellular replication of AB42049, *S*. Typhimurium SL1344, SL1344 *ssaR* isogenic strain and AB42049 expressing *sseJ* or *katE* from a low copy number plasmid (pWSK29) was tested by the gentamicin protection assay in DF-1 chicken fibroblasts. Intracellular replication was determined by the ratio between the numbers of intracellular bacteria at 8 h p.i. relative to their number at 2 h p.i. 1–Way ANOVA with Dunnett’s Multiple Comparison Test was used to determine statistical difference. ***, *P*<0.001.
(TIF)

S1 Table Variant calling between SO7676-03 and AB42049. Variant calling between the genomes of SO7676-03 and AB42049 was conducted by comparing the published genome of SO7676-03 (PRJEB34599) against the short reads of MpSTM AB42049 using Snippy v4.6.0 (https://github.com/tseemann/snippy). The identified changes between these genomes, their, position and the type of change are listed in S1 Table.
(XLSX)

S2 Table Pseudogene prediction in the AB42049 genome. The assembled genome of isolate AB42049 was annotated with DFAST using the published genomes of STM SL1344 (NC_016810) and SO7676-03 (PRJEB34599) as references. DFAST functional annotation includes prediction of pseudogenes as listed in S2 Table.
(XLSX)

S3 Table Strains and plasmids used in this study. All bacterial strains and plasmids used in this study are listed in S3 Table.
(DOCX)

S4 Table Primers used in this study. The oligonucleotide primers used in this study and their DNA sequence are listed in S4 Table.
(DOCX)

S5 Table Genome assemblies of MpSTM from avian origin used to build the phylogenetic tree. 38 genome assemblies of MpSTM isolated from avian or poultry origin that were used to create the phylogenetic tree presented in Fig 1C are listed in S5 Table. All assemblies were downloaded from Enterobase (https://enterobase.warwick.ac.uk/). The genome assemblies and their related metadata are indicated.
(XLSX)

S6 Table Genome assemblies of *S*. Typhimurium isolates from human and avian sources used to calculate the occurrence of pseudogenes found in AB42049. To determine the occurrence of genes of interest in the genomes of *S*. Typhimurium isolates, all of the 2972 genome assemblies of avian isolates available from Enterobase (as for Nov. 2020) and 3134 genome assemblies from clinical (human) origin were selected. All assemblies and their related metadata are listed in S6 Table.
(XLSX)
