# Supplementary figures and images for: Pathoadaptation of the passerine-associated Salmonella enterica serovar Typhimurium lineage to the avian host
Source: PLoS Pathog. 2021 Mar 19;17(3):e1009451. doi: 10.1371/journal.ppat.1009451 (PMC8011750; doi:10.1371/journal.ppat.1009451)

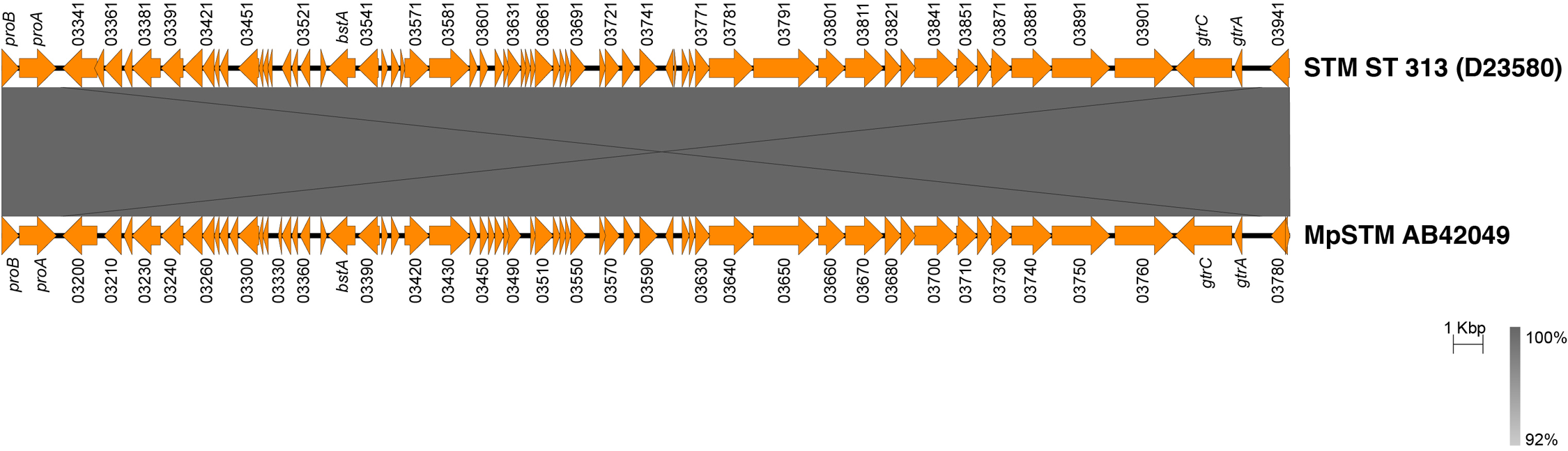

Supplement: S1 Fig — Analysis of the sparrow-associated strain AB42049 genome by PHASTER identified the presence of the S. Typhimurium ST 313 prophage BTP1. Pairwise alignment between the BTP1 region of S. Typhimurium ST313 str. D23580 (NC_016854; position 366797–410321) and the corresponding region of AB42049 (position 365947–409906) is shown. Sequence homology is illustrated by the shades of grey. (TIF) [file ppat.1009451.s001.tif]

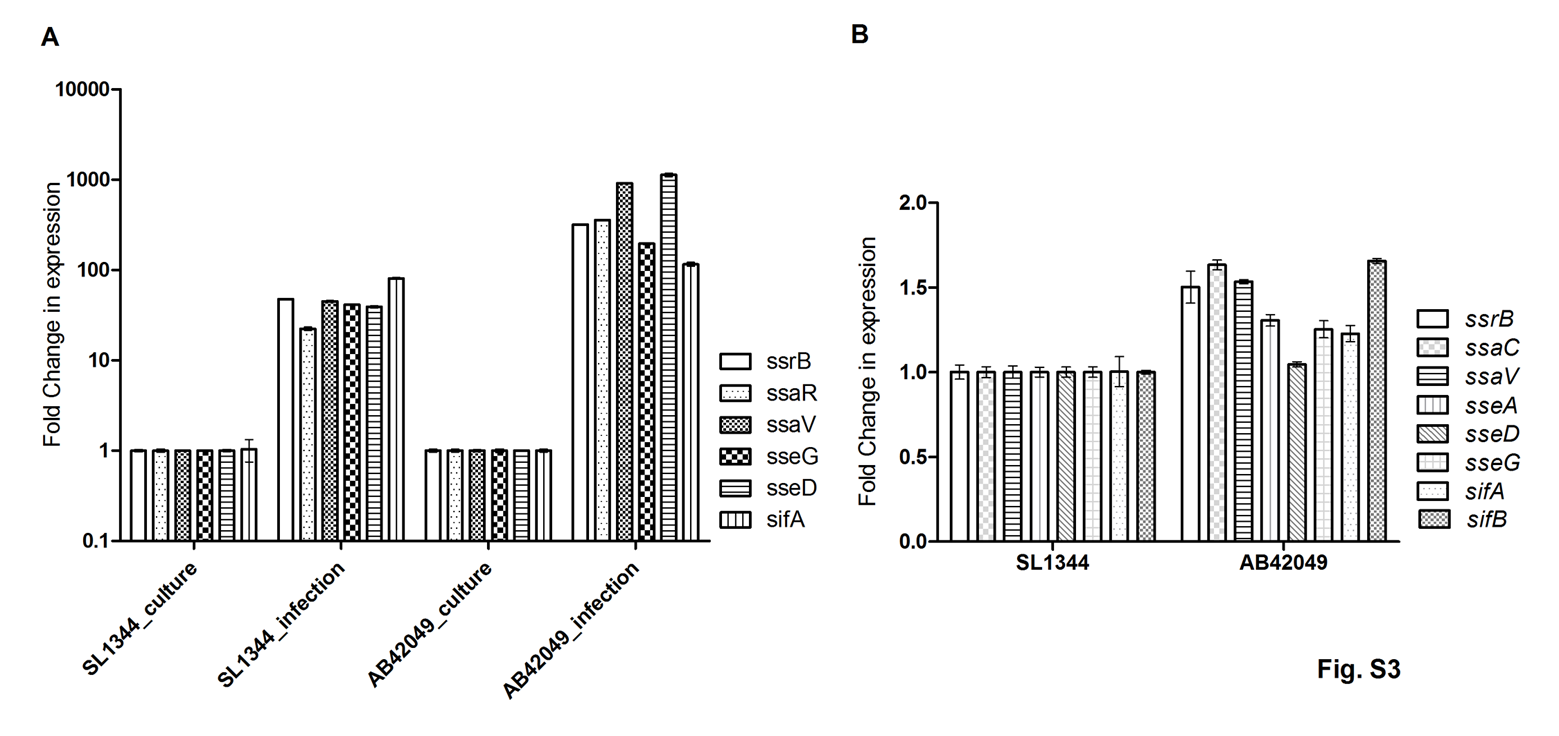

Supplement: S3 Fig — (A) S. Typhimurium SL1344 and AB42049 strains were grown to the late logarithmic phase and used to infect DF-1 cells at MOI of 25. 8 h p.i. RNA was extracted from the infected cells and from the LB grown cultures. qRT-PCR was used to determine the fold change in expression of ssrB, ssaR, ssaV, sseG, sseD, and sifA in intracellular Salmonella vs. their expression in LB grown cultures. The house keeping genes rpoD and 16S rRNA were used for normalization of target genes. The values represent the fold change of the intracellular expression compared to the expression in LB culture. (B) The fold change in the expression of ssrB, ssaC, ssaV, sseA, sseD, sseG, sifA and SifB was determined for intracellular AB42049 relative to the expression of these genes in intracellular S. Typhimurium SL1344 using qRT-PCR. The indicated values show the mean of three repeats and the SEM is represented by the error bars. (TIF) [file ppat.1009451.s003.tif]

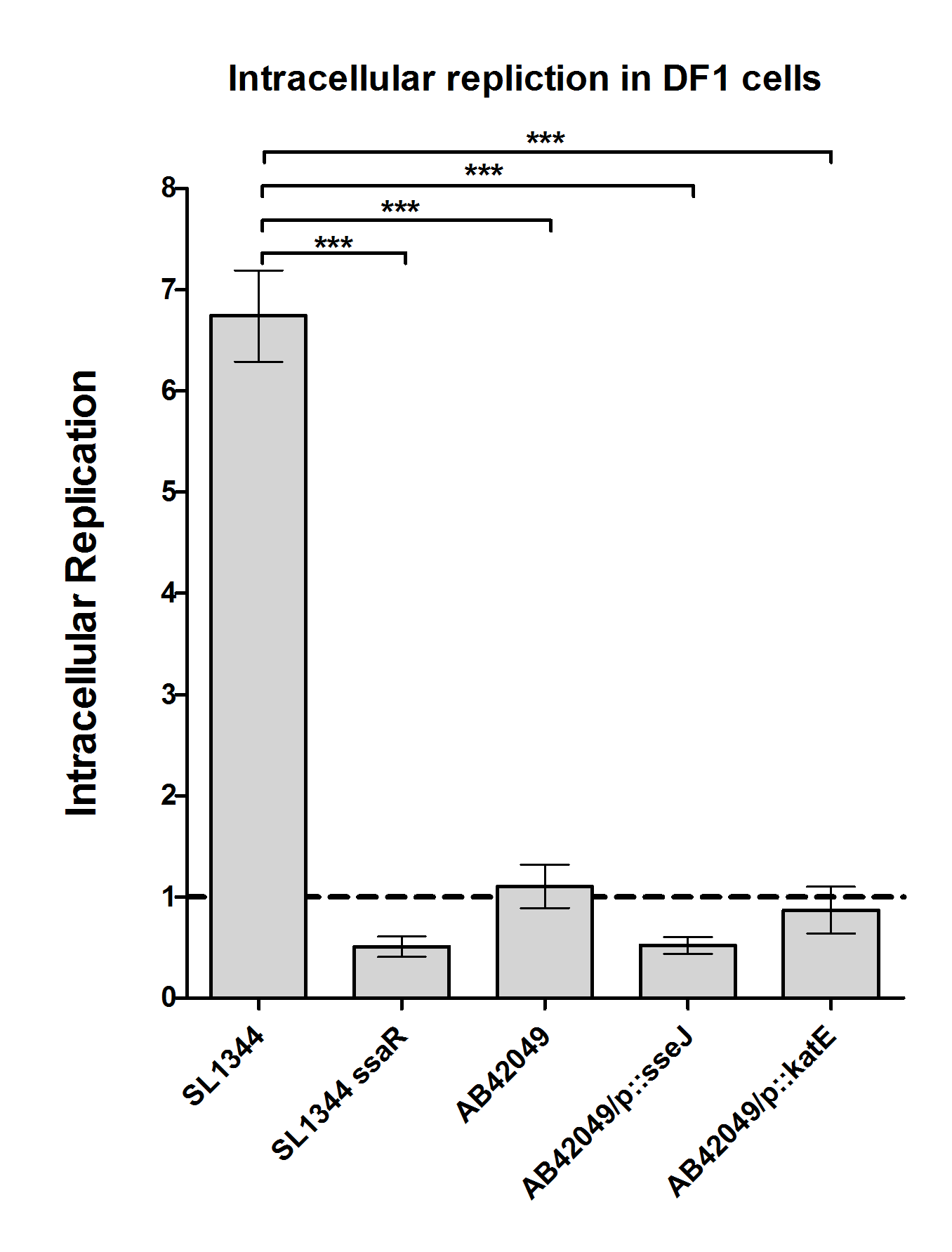

Supplement: S4 Fig — Intracellular replication of AB42049, S. Typhimurium SL1344, SL1344 ssaR isogenic strain and AB42049 expressing sseJ or katE from a low copy number plasmid (pWSK29) was tested by the gentamicin protection assay in DF-1 chicken fibroblasts. Intracellular replication was determined by the ratio between the numbers of intracellular bacteria at 8 h p.i. relative to their number at 2 h p.i. 1–Way ANOVA with Dunnett’s Multiple Comparison Test was used to determine statistical difference. ***, P<0.001. (TIF) [file ppat.1009451.s004.tif]
