# Supplementary material for: Pathoadaptation of the passerine-associated Salmonella enterica serovar Typhimurium lineage to the avian host
Source: PLoS Pathog. 2021 Mar 19;17(3):e1009451. doi: 10.1371/journal.ppat.1009451 (PMC8011750; doi:10.1371/journal.ppat.1009451)
Supplement: S2 Fig — Amino acids alignment of the T3SS-2 effector proteins SseJ (A), SteC (B), SseK2 (C), SseK3 (D), and GogB (D) and the KatE catalase (F) between their wildtype sequence in S. Typhimurium SL1344 and their inactivated sequence in AB42049 is shown. All pseudogenes identified in the AB42049 assembly were confirmed by PCR amplification and Sanger sequencing of the resulted product. (PDF) [file ppat.1009451.s002.pdf]

A. *sseJ* SPI-2 effector gene

```
SL1344      1 MPLSVGQGYFTSSISSEKFNAIKESARLPELSLWEKIKAYFFTTHHAEALECIFNLYHHQ
AB42049     1 MPLSVGQGYFTSSISSEKFNAIKESARLPELSLWEKIKAYFFTTHHAEALECIFNLYHHQ

SL1344     61 ELNLTPVQVRGAYIKLRALASQGCKEQFIIESQEHADKLIIKDDNGENILSIEVECHPEA
AB42049    61 ELNLTPVQVRGAYIKLRALASQGCKEQFIIESQEHADKLIIKDDNGENILSIEVECHPEA

SL1344    121 FGLAKEINKSHPKPKNISLGDITRLVFFGDSLSDSLGRMFEKTHHILPSYGQYFGGRFTN
AB42049   121 FGLAKEINKSHPKPKNISLGDITRLVFFGDSLSDSLGRMFEKTHHILPSYGQYFGGRFTN

SL1344    181 GFTWTEFLSSPHFLGKEMLNFAEGGSTSASYSCFNCIGDFVSNTDRQVASYTPSHQDLAI
AB42049   181 GFTWTEFLSSPHFLGKEMLNFAEGGSTSASYSCFNCIGDFVSNTDRQVASYTPSHQDLAI

SL1344    241 FLLGANDYMTLHKDNVIMVVEQQIDDIEKIISGGVNNVLVMGIPDLSLTPYGKHSDEKRK
AB42049   241 FLLGANDYMTLHKDNVIMVVEQQIDDIEKIISGGVNNVLVMGIPDLSLTPYGKHSDEKRK

SL1344    301 LKDESIAHNALLKTNVEELKEKYPQHKICYYETADAFKVIMEAASNIGYDTENPYTHHGY
AB42049   301 LKDESIAHNALLKTNVEELKEKYPSIKYAITRLPMHLR*---------------------

SL1344    361 VHVPGAKDPQLDICPQYVFNDLVHPTQEVHHCFAIMLESFIAHHYSTE*
AB42049       -------------------------------------------------
```

B. *steC* SPI-2 effector gene

```
SL1344      1 MPFTFQIGNHSCQISERYLRDIIDNKREHVFSTCEKFIDFFRNIFTRRSLISDYREIYNL
AB42049     1 MPFTFQIGNHSCQISERYLRDIIDNKREHVFSTCEKFIDFFRNIFTKRSLISDYREIYNL

SL1344     61 LCQKKEHPDIKGPFSPGPFSKRDEDCTRWRPLLGYIKLIDASRPETIDKYTVEVLAHQEN
AB42049    61 LCQKKRASRY*-------------------------------------------------

SL1344    121 MLLLQMFYDGVLVTETECSERCVDFLKETMFNYNNGEITLAALGNDNLPPSEAGSNGIYE
AB42049       ------------------------------------------------------------

SL1344    181 AFEQRLIDFLTTPATASGYESGAIDQTDASQPAAIEAFINSPEFQKNIRMRDIEKNKIGS
AB42049       ------------------------------------------------------------

SL1344    241 GSYGTVYRLHDDFVVKIPVNERGIKVDVNSPEHRNCHPDRVSKYLNMANDDKNFSRSAIM
AB42049       ------------------------------------------------------------

SL1344    301 NINGKDVTVLVSKYIQGQEFDVEDEDNYRMAEALLKSRGVYMHDINILGNILVKEGVLFF
AB42049       ------------------------------------------------------------

SL1344    361 VDGDQIVLSQESRQQRSVSLATRQLEEQIKAHHMIKLKRAETEGNTEDVEYYKSLITDLD
AB42049       ------------------------------------------------------------

SL1344    421 ALIGEEEQTPAPGRRFKLAAPEEGTLVAKVLKDELKK*
AB42049       --------------------------------------
```

C. *sseK2* SPI-2 effector gene

```
SL1344      1 MARFNAAFTRIKIMFSRIRGLISCQSNTQTIAPTLSPPSSGHVSFAGIDYPLLPLNHQTP
AB42049     1 MARFNAAFTRIKIMFSRIRGLISCQSNTQTIAPTLSPPSSGHVSFAGIDYPLLPLNHQTP

SL1344     61 LVFQWFERNPDRFGQNEIPIINTQKNPYLNNIINAAIIEKERIIGIFVDGDFSKGQRKAL
AB42049    61 LVFQWFERNPDRFGQNEIPIINTQKNPYLNNIINAAIIEKERIIGIFVDGDFSKGQRKAL

SL1344    121 GKLEQNYRNIKVIYNSDLNYSMYDKKLTTIYLENITKLEAQSASERDEVLLNGVVKKSLED
AB42049   121 GKLEQNYRNIKVIYNSDLNYSMYDKKLTTIYLENITKLEAQSASERDEVLLNGVKKAWKM

SL1344    181 VLKNNPEETLISSHNKDKGHLWFDFYRNLFLLKGSDAFLEAGKPGCHHLQPGGGCIYLDA
AB42049   181 S*----------------------------------------------------------
```

```
SL1344    241 DMLLTDKLGTLYLPDGIAIHVSRKDNHVSLENGIIAVNRSEHPALIKGLEIMHSKPYGDP
AB42049       ------------------------------------------------------------

SL1344    301 YNDWLSKGLRHYFDGSHIQDYDAFCDFIEFKHENIIMNTSSLTASSWR*
AB42049       -------------------------------------------------
```

D. *sseK3* SPI-2 effector gene

```
SL1344      1 MFSRVRGFLSCQNYSHTATPAITLPSSGSANFAGVEYPLLPLDQHTPLLFQWFERNPSRF
AB42049     1 MFSRVRGFLSCQNYSHTATPAITLPSSGSANFAGVEYPLLPLDQHTPYFFNGLNETQAGL

SL1344     61 GENQIPIINTQQNPYLNNIINAAIIEKERTIGVLVDGNFSAGQKKALAKLEKQYENIKVI
AB42049    61 GKTRSQLLILNKTPISIILSTPL*------------------------------------

SL1344    121 YNSDLDYSMYDKKLSDIYLENIAKIEAQPANVRDEYLLGEIKKSLNEVLKNNPEESLVSS
AB42049       ------------------------------------------------------------

SL1344    181 HDKRLGHVRFDFYRNLFLLKGSNAFLEAGKHGCHHLQPGGGCIYLDADMLLTGKLGTLYL
AB42049       ------------------------------------------------------------

SL1344    241 PDGIAVHVSRKGNSMSLENGIIAVNRSEHPALKKGLEIMHSKPYGDPYIDGVCGGLRHYF
AB42049       ------------------------------------------------------------

SL1344    301 NCSIRHNYEEFCNFIEFKHEHIFMDTSSLTISSWR*
AB42049       ------------------------------------
```

E. *gogB* SPI-2 effector gene

```
SL1344      1 LTYRLKKRMKIGFQPAILQYAYTSNEATSNLELLNKWRIESPDIEKEERNSIYDKIIEAN
AB42049     1 LTYRLKKRMKIGFQPAILQYAYTSNEATSNLELLNKWRIESPDIEKEERNSIYDKIIEAN

SL1344     61 HTGSLSITAHHVTSIPVFPDNLSELNLSSCYTLESIPNLPDGLKSLTISGNQTIKISYFP
AB42049    61 HTGSLSITAHHVTSIPVFPDNLSELNLSSCYTLESIPNLPDGLKSLTISGNQTIKISYFP

SL1344    121 DSLESLSIDMQAYEENYTFPALPYGLKSFTACYGKFLPPLPPHLSSLSLQNFSEILCAEL
AB42049   121 DSLESLSIDMQAYEENYTFPALPYGLKSFTACYGKFLPPLPPHLSSLSLQNFSEILCAEL

SL1344    181 PYKLDKLDLQNCPFLPLMKMLPEELKELSIELIRTVPGTVIDDILPDKLKKLSINFCDNI
AB42049   181 PYKLDKLDLQNCPFLPLMKMLPEELKELSIELIRTVPGTVIDDILPDKLKKLSINFCDNI

SL1344    241 KLPVKLPVNLKSINLSSRTPIAWEIPTCNLPAHIDISTDGYVKLNPEFLTRSDITFSNKP
AB42049   241 KLPVKLPVNLKSINLSSRTPIAWEIPTCNLPAHIDISTDGYVKLNPEFLTRSDITFSNKP

SL1344    301 AGDVLSFQPGDVVYGLCKARDRVNTLVNSLYYFSKKDIIIQNTLTDAVWDRKNRAVFNKD
AB42049   301 AGDVLSFQPGDVVYGLCKARDRVNTLVNSLYYFSKKDIIIQNTLTDAVWDRKNRAVFNKD

SL1344    361 EKIAERLNDVQRGIFFREFLSQHKKYNITEDKYSDLSNEECWIKTSKAGLEFQTRLRERS
AB42049   361 EKIAERLNDVQRGIFLENFYLNIKNTILPKINIQTYPMRSAG*-----------------

SL1344    421 VIFVIDNLVDAISDIANKTGKHGNSITAHELRWVYRNRHDDLVKQNVKFFLNGEAISHED
AB42049       ------------------------------------------------------------

SL1344    481 VFSLVGWDKYKPKNRNR*
AB42049       ------------------
```

F. *katE* catalase

```
SL1344      1 MSHNEKSPHQSPVHDTRESQPGLDSLAPSDGSHRPTPETTPPGAQPTAPGSLKAPETAND
AB42049     1 MSHNEKSPHQSPVHDTRESQPGLDSLAPSDGSHRPTPETTPPGAQPTAPGSLKAPETAND

SL1344     61 KLTALDAFRKGSENYALTTNQGVRIADDQNSLRAGSRGPTLLEDFILREKITHFDHERIP
AB42049    61 KLTALDAFRKGSENYALTTNQGVRIADDQNSLRAGSRGPTLLEDFILREKITHFDHERIP
```

```
SL1344   121 ERIVHARGSAAHGYFQPYKDLSDITKAAFLCDPQKITPVFVRFSTVQGGAGSADTVRDIR
AB42049  121 ERIVHARGSAAHGYFQPYKNLSDITKAAFLCDPQKITPVFVRFSTVQGGAGSADTVRDIR

SL1344   181 GFATKFYTEEGIFDLVGNNTPIFFIQDAHKFPDFVHAVKPEPHWAIPQGQSAHDTFWDYV
AB42049  181 GFATKFYT*---------------------------------------------------

SL1344   241 SLQPETLHNVMWAMSDRGIPRSYRTMEGFGIHTFRLINAQGKATFVRFHWKPLAGKASLV
AB42049      ------------------------------------------------------------

SL1344   301 WDESQKLTGRDPDFHRRDLWEAIEAGDFPEYELGLQLIAEEDEFKFDFDLLDPTKLIPEE
AB42049      ------------------------------------------------------------

SL1344   361 LVPVQRVGKMVLNRNPDNFFAENEQAAFHPGHIVPGIDFTNDPLLQGRLFSYTDTQISRL
AB42049      ------------------------------------------------------------

SL1344   421 GGPNFHEIPINRPTCPYHNFQRDGMHRMDIDTNPANYEPNSINDNWPRETPPAPKRGGFE
AB42049      ------------------------------------------------------------

SL1344   481 SYQERVDGNKIRERSPSFGEYYSHPRLFWLSQTPIEQQHIIDAFSFELGKVARAYIRERV
AB42049      ------------------------------------------------------------

SL1344   541 VDQLAHIDVTLAQGVAHNLGFALTHEQTQIAPPPDVNGLKKDPALSLYAVPDGDVKGRVV
AB42049      ------------------------------------------------------------

SL1344   601 AILLNDKVNAAELLTILQALKAKGVHAKLLYSRMGEVTADDGSTLTIAATFAGAPSLTVD
AB42049      ------------------------------------------------------------

SL1344   661 AVIVPCGNIADIESCGDARYYLLEAYKHLKPIALAGDARRFKALLNIDSQGEEGLVEADN
AB42049      ------------------------------------------------------------

SL1344   721 VDHHFMDTLLTLMAAHRVWSRAGKINAIPA*
AB42049      ------------------------------
```

Fig. S2
