# Supplementary material for: Pathoadaptation of the passerine-associated Salmonella enterica serovar Typhimurium lineage to the avian host
Source: PLoS Pathog. 2021 Mar 19;17(3):e1009451. doi: 10.1371/journal.ppat.1009451 (PMC8011750; doi:10.1371/journal.ppat.1009451)
Supplement: S3 Table — All bacterial strains and plasmids used in this study are listed in S3 Table. (DOCX) [file ppat.1009451.s007.docx]

**Table S3. Strains and plasmid used in this study**

| **Strains** | **genotype/ description** | **Source** |
| --- | --- | --- |
| *S*. Typhimurium SL1344 | wild type Sm^r^ *xyl hisG rpsL* | SGSC^†^ |
| *S*. Typhimurium SL1344 invA | Δ*invA* | [1] |
| *S*. Typhimurium SL1344 ssaR | Δ*ssaR* | [2] |
| *S*. Typhimurium SL1344 katE | Δ*katE* | This study |
| monophasic *S*. Typhimurium AB42049 | Sparrow-associated strain | isolated in Israel 2015. accession number CP064919 |
| monophasic *S*. Typhimurium AB42052 | Sparrow-associated strain | isolated in Israel 2015  accession number CP064918 |
| monophasic *S*. Typhimurium AB42086 | Sparrow-associated strain | isolated in Israel 2015  accession number CP064917 |
| monophasic *S*. Typhimurium AB42142 | Sparrow-associated strain | isolated in Israel 2015  accession number CP064916 |
| *S*. Gallinarum 287/91 | Poultry-adopted serovar | SGSC^†^ |
| **Plasmids** | | |
| p3776 (PssaG::sfGFP) | amp^r^ pEM7::TagRFP-T PssaG::sfGFP | [3] |
| p4889 (PuhpT::sfGFP) | amp^r^ pEM7::DsRed PuhpT::sfGFP | [4] |
| Ptet::sfGFP | amp^r^ pEM7:TagRFP-T PtetA::sfGFP | [5] |
| pWSK29::katE | amp^r^ *katE* | this study |
| pWSK29::sseJ | amp^r^ *sseJ* | this study |
| pWSK29 | amp^r^ | [6] |
| pKD4 | amp^r^ kan^r^ | [7] |
| pKD46 | amp^r^ | [7] |
| pCP20 | amp^r^ cm^r^ | [8] |

^†^ Salmonella Genetic Stock Centre - University of Calgary.

**REFERENCES**

1. Galan JE, Curtiss R, 3rd. Distribution of the invA, -B, -C, and -D genes of Salmonella typhimurium among other Salmonella serovars: invA mutants of Salmonella typhi are deficient for entry into mammalian cells. Infect Immun. 1991;59(9):2901-8. Epub 1991/09/01. PubMed PMID: 1879916; PubMed Central PMCID: PMC258111.

2. Brumell JH, Rosenberger CM, Gotto GT, Marcus SL, Finlay BB. SifA permits survival and replication of Salmonella typhimurium in murine macrophages. Cell Microbiol. 2001;3(2):75-84. Epub 2001/02/24. PubMed PMID: 11207622.

3. Röder J, Hensel M. 2020. doi: 10.1101/2020.10.23.351551.

4. Noster J, Chao TC, Sander N, Schulte M, Reuter T, Hansmeier N, et al. Proteomics of intracellular Salmonella enterica reveals roles of Salmonella pathogenicity island 2 in metabolism and antioxidant defense. PLoS Pathog. 2019;15(4):e1007741. Epub 2019/04/23. doi: 10.1371/journal.ppat.1007741. PubMed PMID: 31009521; PubMed Central PMCID: PMCPMC6497321.

5. Schulte M, Sterzenbach T, Miskiewicz K, Elpers L, Hensel M, Hansmeier N. A versatile remote control system for functional expression of bacterial virulence genes based on the tetA promoter. Int J Med Microbiol. 2019;309(1):54-65. Epub 2018/12/07. doi: 10.1016/j.ijmm.2018.11.001. PubMed PMID: 30501934.

6. Wang RF, Kushner SR. Construction of versatile low-copy-number vectors for cloning, sequencing and gene expression in *Escherichia coli*. Gene. 1991;100:195-9. Epub 1991/04/01. PubMed PMID: 2055470.

7. Datsenko KA, Wanner BL. One-step inactivation of chromosomal genes in Escherichia coli K-12 using PCR products. Proc Natl Acad Sci U S A. 2000;97(12):6640-5. Epub 2000/06/01. doi: 10.1073/pnas.120163297. PubMed PMID: 10829079; PubMed Central PMCID: PMCPMC18686.

8. Cherepanov PP, Wackernagel W. Gene disruption in Escherichia coli: TcR and KmR cassettes with the option of Flp-catalyzed excision of the antibiotic-resistance determinant. Gene. 1995;158(1):9-14. Epub 1995/05/26. doi: 10.1016/0378-1119(95)00193-a. PubMed PMID: 7789817.
