# Supplementary material for: Pathoadaptation of the passerine-associated Salmonella enterica serovar Typhimurium lineage to the avian host
Source: PLoS Pathog. 2021 Mar 19;17(3):e1009451. doi: 10.1371/journal.ppat.1009451 (PMC8011750; doi:10.1371/journal.ppat.1009451)
Supplement: S4 Table — The oligonucleotide primers used in this study and their DNA sequence are listed in S4 Table. (DOCX) [file ppat.1009451.s008.docx]

**Table S4. Primers used in this study**

| **#** | **Primer name** | **sequence (5'-3')** | **Usage** |
| --- | --- | --- | --- |
| 1 | invA-F | TTC ACT GAC TTG CTA TCT GC | Confirming *Salmonella* spp. identification |
| 2 | InvA-R | TCA GGA AAC AAA ACA TAT GC |  |
| 3 | stec_Fwd | CGCCATCGTGTACAGTCTTC | PCR and Sanger sequencing for validation of psuedogene |
| 4 | sreC_Rev | TCATCCAACCAGAGACACCG |  |
| 5 | ssek2_Fwd | GGCGATTTTAGCAAGGGACA | PCR and Sanger sequencing for validation of psuedogene |
| 6 | ssek2_rev | GGTTTACCAGCTTCCAGAAA |  |
| 7 | ssek3_fwd | CTGTCCCCCTGCAATACAAT | PCR and Sanger sequencing for validation of psuedogene |
| 8 | sseK3_rev | CGTTTATCATGGGACGAAAC |  |
| 9 | gogB_Fwd | TCAGCCTGGAGATGTGGTTT | PCR and Sanger sequencing for validation of psuedogene |
| 10 | gogB_Rev | CCGTTCCCTCAATCGTGTTT |  |
| 11 | OA003F | TTTTctcgagGCTGGCGAAGAGGTTTCATC | Cloning of S. Typhimurium catalase *katE* into pWSK29 (XhoI) |
| 12 | OA003R | TTTTtctagaTGTTGACGTCTGCGTCACTC | Cloning of S. Typhimurium catalase *katE* into pWSK29 (XbaI) |
| 13 | OA006Fseq1 | TACTGGGATAGGCTCTAAG | Sanger sequencing of pWSK29/ *katE* |
| 14 | OA006Fseq2 | GCCGTCAGACGGTTCACATC | Sanger sequencing of pWSK29/ *katE* |
| 15 | OA006Fseq3 | AACGTCATGTGGGCAATGTC | Sanger sequencing of pWSK29/ *katE* |
| 16 | OA006Fseq4 | CGAACAGGCGGCATTTCATC | Sanger sequencing of pWSK29/ *katE* |
| 17 | OA006Fseq5 | AGACGCCGATAGAACAGCAG | Sanger sequencing of pWSK29/ *katE* |
| 18 | OA006Fseq6 | AACGGTCGATGCGGTAATAG | Sanger sequencing of pWSK29/ *katE* |
| 19 | OA004F | TTTTtctagaTCAAGCCAGCTCATACTCAC | Cloning of S. Typhimurium *sseJ* into pWSK29 (XbaI) |
| 20 | OA004R | TTTTgagctcCCGGCACTATGATATTGAGC | Cloning of S. Typhimurium *sseJ* into pWSK29 (SacI) |
| 21 | katE_P1 | CAGTGATGAAAGCAGGAGACGAGTTCAATGTCGCATAATGAGAAAGTGTAGGCTGGAGCTGCTTCG | Deletion of *katE* |
| 22 | katE_P2 | GCAAGAATGCGCCGCATTTTTTGCTGTTTATGCAGGAATCGCGTTCATATGAATATCCTCCTTA |  |
| 23 | katE_5'_flank | TAACGCCTACTGGGATAG | *katE* deletion verification |
| 24 | katE_3'_flank | GAGATCACCGAAGCGTGC |  |
| 25 | spvB F | TCCGTCAGACCCGTAAACAGT | validation of pSLT absence |
| 26 | spvB R | GCTGAGCGTTTGAACCAGC |  |
| 27 | GAPDH Fw (chicken) | CGCCATCACTATCTTCCAGGAG | qRT-PCR of *Gapdh* in chickens |
| 28 | GAPDH Rv (chicken) | GGAGCTGAGATGATAACACGCTTAG |  |
| 29 | cIL1b-RT-F | GCTCTACATGTCGTGTGTGATGAG | qRT-PCR of *IL1B* in chickens |
| 30 | cIL1b-RT-R | TGTCGATGTCCCGCATGA |  |
| 31 | cIL6-RT-F | GCTCGCCGGCTTCGA | qRT-PCR of *IL6* in chickens |
| 32 | cIL6-RT-R | GGTAGGTCTGAAAGGCGAACAG |  |
| 33 | cIFNg-RT-F | GCCGCACATCAAACACATATCT | qRT-PCR of *IFNG* in chickens |
| 34 | cIFNg-RT-R | TGAGACTGGCTCCTTTTCCTT |  |
| 35 | cIL18-RT-F | TCT GGC AGT GGA ATG TAC TTC G | qRT-PCR for *IL18* in chickens |
| 36 | cIL18-RT-R | CCA TTT TCC CAT GCT CTT TCT C |  |
| 37 | cNOS2-RT-F | GAA CAG CCA GCT CAT CCG ATA | qRT-PCR for *NOS2* in chickens |
| 38 | cNOS2-RT-R | CCC AAG CTC AAT GCA CAA CTT |  |
| 39 | IL-22_chk_F | CAG ACT CAT CGG TCA GCA AA | qRT-PCR for *IL22* in chickens |
| 40 | IL-22_chk_R | GGT ACC TCT CCT TGG CCT CT |  |
| 41 | RT 16s rRNA Fw | GGTTAAGTCCCGCAACGAG | qRT-PCR for 16S rRNA in Salmonella |
| 42 | RT 16s rRNA Rv | CTTCTCTTTGTATGCGCCATTG |  |
| 43 | rpoD Fw | GGTCTGACCATCGAACAGGTG | qRT-PCR for *rpoD* in Salmonella |
| 44 | rpoD Rev | ATCAGACCGATGTTGCCTTC |  |
| 45 | ssaR(yscR)-RT F | ATGTCTTCAGGCCAGGTTCG | qRT-PCR for *ssaR* in Salmonella |
| 46 | ssaR(yscR)-RT F | TTCTGGACGTCTGAGTGGG |  |
| 47 | ssrB-RT F | TGGCCTGGATATCATTCCTC | qRT-PCR for *ssrB* in Salmonella |
| 48 | ssrB-RT R | TTGCAATGCCGCTAACAGAAC |  |
| 49 | sseA_F | GTG CCG AGA AAG TGT CCA TT | qRT-PCR for *sseA* in Salmonella |
| 50 | sseA_R | CCT CAA ACT CGC CTC CTT CA |  |
| 51 | sseD_F | TGC CGA CCT GCA ACA AAA TG | qRT-PCR for *sseD* in Salmonella |
| 52 | sseD_R | TTC AGC AAG AAC CGT GAC CA |  |
| 53 | sifB_F | CCG CTA TGT TGC TTG TTC CC | qRT-PCR for *sifB* in Salmonella |
| 54 | sifB_R | TCC TGT TCC TTC CAG TCA CAT |  |
| 55 | sseG_F | ACT CGC TTC GGT ATG GAT GG | qRT-PCR for *sseG* in Salmonella |
| 56 | sseG_R | CGT TGT TCT GGC GTT ACC TG |  |
| 57 | ssaV_F | ATC TCA CCG TTG GGT TGG TC | qRT-PCR for *ssaV* in Salmonella |
| 58 | ssaV_R | CTA ACT TCC GCC ACC CTC TC |  |
| 59 | ssaC_F | CCG GGA TGC GAG GTT AAA GA | qRT-PCR for *ssaC* in Salmonella |
| 60 | ssaC_R | CTC ACC GCA CTG TCT TTT CG |  |
| 61 | sifA-RT_F | CAACTCCCCAAGGAATACG | qRT-PCR for *sifA* in Salmonella |
| 62 | sifA-RT_R | ATCTCTGTAAGCCGCTCTCG |  |
| 63 | mIFNgama RT F | TCAAGTGGCATAGATGAAGAA | qRT-PCR for *Ifng* in mice |
| 64 | mIFNgama RT R | TGGCTCTGCAGGATTTTCATG |  |
| 65 | mIL-1b RT F | AACCTGCTGGTGTGTGACGTTC | qRT-PCR for *Il1b* in mice |
| 66 | mIL-1b RT R | CAGCACGAGGCTTTTTTGTTGT |  |
| 67 | m TNF alfa RT F | TGGCCCAGACCCTCACACTCAG | qRT-PCR for *Tnfa* in mice |
| 68 | m TNF alfa RT R | ACCCATCGGCTGGCACCACT |  |
| 69 | mIL-6 RT F | GAGGATACCACTCCCAACAGACC | qRT-PCR for *Il6* in mice |
| 70 | mIL-6 RT R | AAGTGCATCATCGTTGTTCATACA |  |
| 71 | mGAPDH RT F | ATTGTCAGCAATGCATCCTG | qRT-PCR for *Gapdh* in mice |
| 72 | mGAPDH RT R | ATGGACTGTGGTCATGAGCC |  |
